# GMP Manufacturing and Characterization of the HIV Booster Immunogen HxB2.WT.Core-C4b for Germline Targeting Vaccine Strategies

**DOI:** 10.3390/vaccines13090980

**Published:** 2025-09-18

**Authors:** Sammaiah Pallerla, Latha Kallur Siddaramaiah, Philipp Mundsperger, Dietmar Katinger, Katharina Fauland, Günter Kreismayr, Robert Weik, Onur Arslan, Mingchao Shen, Gabriel Ozorowski, Wen-Hsin Lee, Andrew B. Ward, Sabyasachi Baboo, Jolene K. Diedrich, John R. Yates, James C. Paulson, Tracy Blumen, Daniel Craig, Ryan Swoyer, Maoli Yuan, Leonidas Stamatatos

**Affiliations:** 1 IAVI, 125 Broad Street, 9th Floor, New York, NY 10004, USArswoyer@iavi.org (R.S.);; 2 Vaccine and Infectious Disease Division, Fred Hutchinson Cancer Center, 1100 Fairview Ave. N., Seattle, WA 98109, USA; mshen@fredhutch.org (M.S.); lstamata@fredhutch.org (L.S.); 3 Polymun Scientific Immunbiologische Forschung GmbH, Donaustr. 99, 3400 Klosterneuburg, Austriaonur.arslan@polymun.com (O.A.); 4 Department of Integrative Structural and Computational Biology, The Scripps Research Institute, La Jolla, CA 92037, USA; 5 Department Immunology and Microbiology, The Scripps Research Institute, La Jolla, CA 92037, USA

**Keywords:** HIV vaccines, clinical trial material, GMP

## Abstract

Background/Objectives: Despite progress in antiretroviral therapy, HIV remains a major global health challenge with over one million new infections annually. An effective vaccine is urgently needed. Germline-targeting immunogens show promise in initiating broadly neutralizing antibody (bNAb) precursors. This study developed a scalable, cGMP-compliant process to manufacture the HIV vaccine booster immunogen HxB2.WT.Core-C4b, a nanoparticle designed to direct bNAb precursor maturation after priming. Methods: A CHO cell platform was established through single-cell cloning from a high-producing stable pool. Upstream and downstream processes were optimized for scalability and yield. Three scales were tested 10 L, 40 L, and 400 L. Key parameters (pH, temperature, feeding, metabolite profiles) were systematically refined. Analytical characterization included glycosylation profiling, electron microscopy, and antigenicity testing. Viral clearance was evaluated per ICH Q5A guidelines. Results: Optimization ensured consistent yields above 130 mg/L, with titers up to 250 mg/L. The selected clone (4E22) demonstrated strong growth, viability, and reproducibility. Glycan occupancy at 18 N-linked sites, including bNAb epitopes (N276, N332), was stable across scales. Over 70% of self-assembling nanoparticle were fully assembled at the GMP level. Antigenicity and purity met cGMP release criteria. Viral clearance achieved >13-log reduction for enveloped and >7-log for non-enveloped viruses. Conclusions: This work establishes a robust, scalable platform for HIV nanoparticle immunogens. Consistent quality and yield across scales support clinical development of HxB2.WT.Core-C4b and provide a model for other glycosylated nanoparticle vaccines. The immunogen is being evaluated in clinical study HVTN 320 (NCT06796686), enabling early testing of next-generation vaccines designed to elicit broadly neutralizing antibodies.

## 1. Introduction

Human immunodeficiency virus (HIV) remains a persistent global health threat, with an estimated 39.9 million people living with the virus and approximately 630,000 HIV-related deaths recorded in 2023 alone [1]. Despite substantial progress in antiretroviral therapy (ART) and prevention strategies, more than one million new infections continue to occur annually, underscoring the urgent need for an effective vaccine. ART has transformed HIV from a fatal disease into a manageable chronic condition; however, it requires lifelong adherence, poses economic and logistical challenges, and does not eliminate the risk of viral transmission or latent reservoir reactivation [2].

The development of an HIV vaccine has been a major scientific objective, and multiple approaches have been investigated, including subunit proteins, viral vectors, and DNA- and mRNA-based platforms, with varying levels of success [3,4,5]. Notably, recent advances in germline-targeting immunogen design have demonstrated feasible approaches for guiding B cell responses toward broadly neutralizing antibody (bNAb) precursors [6,7,8,9,10]. The HxB2.WT.Core-C4b self-assembling nanoparticle immunogen evaluated in this study was designed as a nanoparticle-displayed, heterologous booster for the germline-targeting priming immunogen 426c.Mod.Core-C4b. The HxB2.WT.Core-C4b that is glycosylated at the conserved N-linked glycosylation site at position 276 in Loop D and at position 463 in V5, does not activate naïve VRC01-class B cells, but plays a crucial role in guiding antibody maturation efficiently by selecting and expanding key somatic mutations when employed as a first booster, facilitating downstream development of bNAbs [10,11,12].

The selection of HxB2.WT.Core-C4b as a “booster” immunogen is based on extensive preliminary experimentation [10,11,12]. We have shown that we can efficiently activate naïve B cells expressing germline (unmutated) VRC01-class BCRs in vivo, by employing the germline-targeting immunogen 426c.Mod.Core-C4b. This activation leads to the accumulation of somatic mutations in the VRC01-class BCRs. Some of these mutations allow the partially mutated BCRs to bypass the N276-associated glycans in Loop D, which represent the major obstacle in the activation and maturation of VRC01-class antibodies. HxB2.WT.Core-C4b is optimal in selecting the BCRs with these binding properties (even though itself does not activate naïve VRC01-class B cells) and furthering their maturation through the accumulation of additional somatic mutations [13,14]. The accumulation of additional somatic mutations will accelerate the elicitation of protective broadly neutralizing VRC01-class antibodies through vaccination.

Parallel to antigen design, manufacturing technologies for HIV vaccines must meet current good manufacturing practices (cGMP) standards while supporting flexibility, scalability, and cost-efficiency; this is especially critical for low- and middle-income countries (LMIC). While mRNA and viral vector vaccines have demonstrated rapid scalability, particularly during the COVID-19 pandemic, protein-based nanoparticle vaccines offer advantages in thermostability, safety profile, and the potential for multivalent display [15,16]. However, scalable and regulatory-compliant production of nanoparticle-based immunogens remains a technical hurdle, such as batch-to-batch consistency, assembly efficiency, and high yields.

This article provides a comprehensive assessment of a scalable, cGMP-compatible process for producing the nanoparticle-based HIV vaccine candidate HxB2.WT.Core-C4b. Initial collaborative work on Chinese hamster ovary (CHO) stable pool cell line development to generate an MCB and process development for manufacturing was conducted by the Duke Human Vaccine Institute (DHVI). It demonstrated effective cell culture media for upstream processes and established purification columns and filtration systems for downstream processing. Building on this, our study describes the next critical steps, including the establishment of a high-yield monoclonal CHO cell line, optimization of upstream and downstream processes, and successful 40-fold scale-up to achieve a cGMP-compliant manufacturing process. This work aims to support the cost-effective and accelerated development of heterologous boosting immunogens for germline-targeting HIV vaccines, providing a template for the broader application of nanoparticle technologies in infectious disease immunization programs.

## 2. Materials and Methods

### 2.1. Stable Clone Generation

A previously generated MCB based on a stable cell pool of HxB2.WT.Core-C4b-expressing CHO cells was subjected to single-cell subcloning by limiting dilution in 384-well plates (Corning). Clonality of individual wells was confirmed using a Solentim Cell Metric automated plate imaging system (Advanced Instruments), and clones with robust growth were expanded stepwise from microplates to shake flasks (Corning). Product expression was assessed by ELISA at the 96-well stage, and the clones with the highest titers were further characterized.

Top-performing subclones were adapted to agitated culture conditions in 50 mL TubeSpin^®^ Bioreactor tubes (TPP) and evaluated for cell growth, viability, and productivity across multiple passages. Cell-specific productivity (qp) and growth rate (µ) were determined, and selected subclones were cryopreserved as research cell banks (RCBs). From these, the three highest-performing clones (designated CHO/HXB2C/4E22/RCB, CHO/HXB2C/9K21/RCB, and CHO/HXB2C/5C9/RCB) were advanced to bioreactor-scale evaluation. Comparative analysis of productivity and product quality identified clone 4E22 as the most promising candidate. Based on these results, a new master cell bank (CHO/HXB2C/4E22/MCB) was established from clone 4E22 and used for process verification and subsequent cGMP manufacturing runs.

### 2.2. Inoculum Preparation and Bioreactor Operation

Inoculum preparation began by thawing RCB or MCB vials of liquid nitrogen cryopreserved cell stocks (1 × 10^7^ cells per vial) under a laminar hood, transfer of the entire cell stock (~1 mL) to 10 mL of cold OptiCHO medium (2–8 °C), and then centrifuging at 170 ± 10 g for 10 min. The supernatant was then discarded in order to remove residual freezing medium and cell pellets were resuspended in pre-warmed (37 °C) OptiCHO medium, transferred to 125 mL shake flasks (Corning), and cultured in an incubator shaker (Kuhner) at 125 rpm, 37 °C, and 5% CO_2_. Inoculum cultures for the bioreactors were prepared through periodic passaging of cultures every 3–4 days including monitoring of cell growth and viability as described under Section 2.6.1 and gradually scaling up to 1 L shake flasks. Once a sufficiently high total cell count was obtained for inoculation of bioreactors at a starting cell density of ≥0.5 × 10^6^ cells/mL, cultures were then transferred to glass bottles for sterile connection to the bioreactor system.

Bioreactor cultivation was carried out in continuous stirred-tank bioreactors, using R’ALF bioreactors (Bioengineering) with total volumes of 6–7 L (working volume 2–4.5 L) or 15 L (working volume 6–10 L). Each bioreactor was equipped with probes for pH, pO_2_, and temperature, along with aeration via headspace, sparger, or microsparger. The process control system managed all parameters. pH was monitored using gel electrodes (Mettler Toledo), initially set at 7.00 and later adjusted to 6.95 or 6.80, with a maximum dead band control of ±0.15 units. A proportional–integral (PI) control loop was used to regulate the addition of corrective CO_2_, delivered either through direct sparging or by adding 1 M sodium carbonate (Na_2_CO_3_). Dissolved oxygen levels (pO_2_) were monitored with amperometric electrodes (Mettler Toledo). For routine cultivation, pO_2_ was maintained at 40% air saturation using a PI control loop by sparging pure oxygen into the culture, supplementing the baseline gas mixture.

The temperature was maintained at 37 ± 0.2 °C, and the stirrer speed was adjusted according to the reactor size, with a standard setting of 150 rpm for R’ALF cultivations. No antifoam solution was needed due to minimal foaming. Cells were inoculated into OptiCHO basal media (supplemented with 4 mM L-glutamine, without methotrexate (MTX)) at a density of ≥5 × 10^5^ cells/mL, with daily addition of 3 × Efficient Feed ™ A + at 1% of the initial working volume from day 3 to 12. The glucose concentration was maintained at ≥2 g/L and supplemented as needed. Developmental adaptations included the use of glucose-enriched feed (120 g/L) to eliminate the need for a separate feed line. Cultures were terminated and harvested at ≥80% viability, targeting a 14-day process time.

### 2.3. Harvest Operation

Harvest clarification was initiated on day 14 or earlier if viability dropped below 80%. Centrifugation (Heraeus Megafuge 40, Hanau, Germany) was carried out at 3000–4500× *g* for 10 min, followed by depth filtration and then 0.45 µm and 0.2 µm filtrations. Filters were rinsed with purified water and buffer solution (20 mM HEPES, 100 mM NaCl, pH 7.2) before use. The optimization of the filtration step involved using an alternative depth filter material, 60ZB05A (3 M), and adding a MAQ absorber (Emphaze, 3 M) before sterile filtration.

### 2.4. Downstream Purification of Recombinant HxB2.WT.Core-C4b Drug Substance Figure 1

#### 2.4.1. Clarification

Harvested cell culture fluid was clarified in a closed system using a CSA1 centrifuge (Westfalia Separator GmbH, Oelde, Germany) operated at 9400–10,000 rpm with a flow rate of 0.5–6 L/min. The centrifuged material was passed sequentially through a cellulose depth filter followed by a 0.2 µm protective filter to remove residual cells and debris. The clarified harvest was then directed through an 3M™ Emphaze™ AEX Series Hybrid Purifier anion exchange membrane adsorber (3M, Saint Paul, MN, USA) operated in flow-through mode to reduce residual host cell DNA.

**Figure 1:**
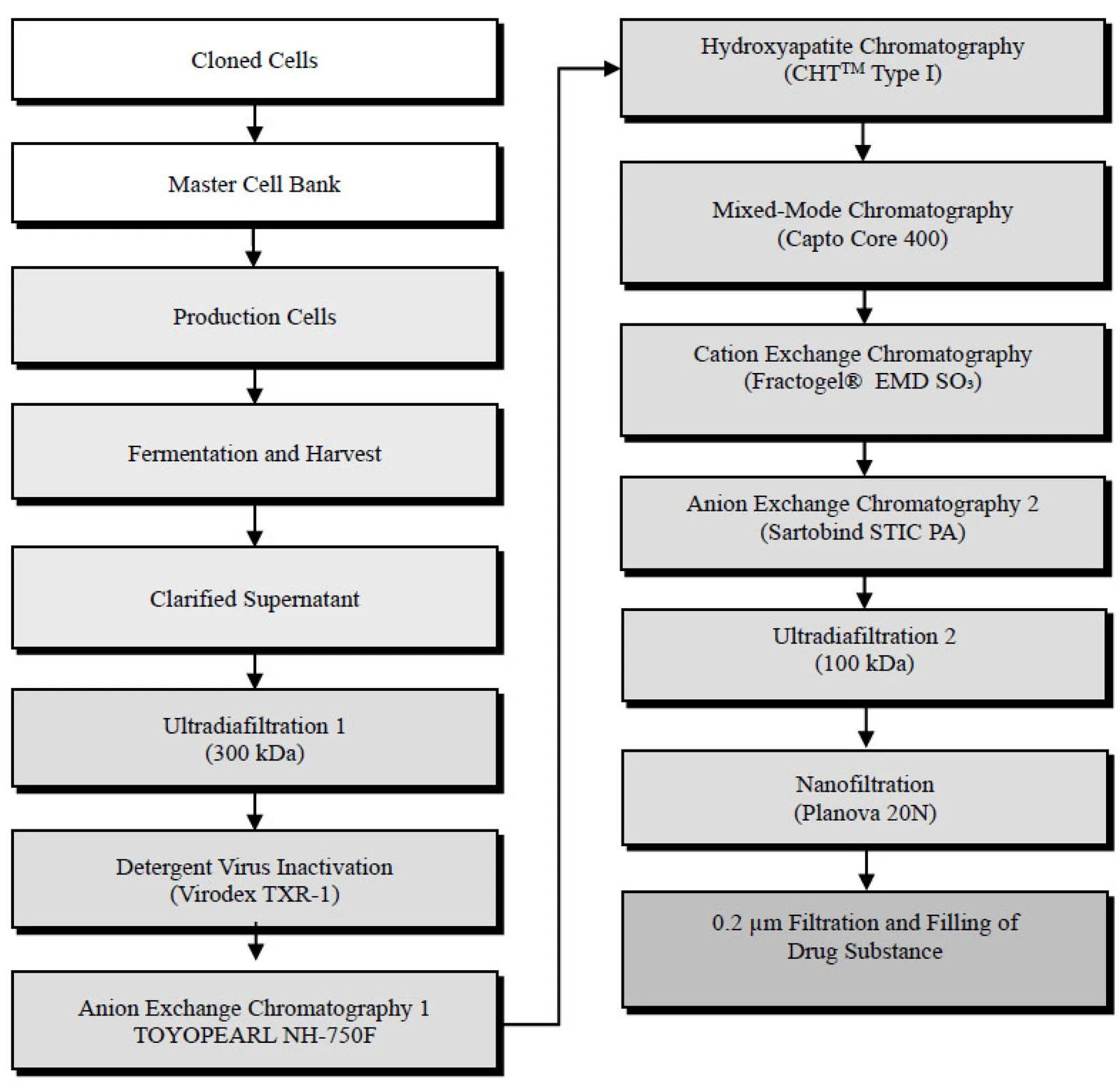
Schematic overview of the cGMP-compliant manufacturing process for HxB2.WT.Core-C4b immunogen production.

#### 2.4.2. Ultradiafiltration

Clarified harvest was concentrated using a crossflow ultrafiltration system fitted with a 300 kDa MWCO cellulose acetate membrane. The retentate was concentrated 5- to 10-fold, followed by diafiltration with at least 10 diavolumes of diafiltration buffer. The retentate was continuously filtered across a 0.2 µm membrane during collection to remove potential particulates. System preparation included flushing with water and sanitization with sodium hydroxide prior to use.

#### 2.4.3. Virus Inactivation (Step 5)

To ensure viral safety, the concentrated product was treated with Virodex TXR1-LQ-(MV) detergent at a final concentration of 0.001 (mass fraction). The mixture was incubated at ≥18 °C for 1–2 h with gentle stirring to achieve effective virus inactivation.

#### 2.4.4. Anion Exchange Chromatography 1

Virus-inactivated material was loaded onto a 25 cm ID column packed with TOYOPEARL NH2-750F resin (Tosoh Bioscience, Tokyo, Japan). The column was equilibrated with buffer at pH 7.0–7.4 and conductivity 23–27 mS/cm, and loading was performed at 160–200 cm/h. After loading, the column was washed with 5 CV equilibration buffer, and the bound protein was eluted with a defined elution buffer. Regeneration was carried out with 2 M NaCl and 0.5 M NaOH, and the column was stored in 20% ethanol when not reused within 24 h.

#### 2.4.5. Hydroxyapatite Chromatography

The anion exchange eluate was subjected to hydroxyapatite chromatography using CHT Type I, 40 µm resin (Bio-Rad, Hercules, CA, USA) packed in an 18 cm ID column. The column was equilibrated with phosphate buffer at pH 7.0–7.4 and conductivity 80–90 mS/cm. Loading was performed at 160–200 cm/h, followed by washes with equilibration buffer and a secondary wash buffer. Protein was eluted with phosphate-based elution buffer, and the column was regenerated with phosphate-based regeneration buffer and stored in sodium hydroxide solution if not reused.

#### 2.4.6. Mixed-Mode Chromatography

The hydroxyapatite eluate was diluted to 0.5 mg/mL and loaded onto a 7 cm ID column packed with Capto Core 400 resin Cytiva, Marlborough, MA, USA). The column was operated in non-binding, flow-through mode at 160–200 cm/h. Flow-through fractions containing the product were collected and the column was regenerated with regeneration buffer before storage in either 0.01 M NaOH or 20% ethanol.

#### 2.4.7. Cation Exchange Chromatography

Flow-through fractions from mixed-mode chromatography were loaded onto a 12.7 cm ID column packed with Millipore Fractogel SO3 resin (Merck KGaA, Darmstadt, Germany). The column was equilibrated with a low-salt buffer at pH 7.0–7.4 and conductivity 11–15 mS/cm, loaded at 160–200 cm/h, and washed sequentially with equilibration and wash buffers. Bound protein was eluted with high-salt elution buffer, and the column was regenerated with 2 M NaCl and stored in ethanol-containing buffer.

#### 2.4.8. Anion Exchange Chromatography 2

The cation exchange eluate was diluted to reduce conductivity and applied to a Sartobind STIC PA membrane adsorber (Sartorius, Göttingen, Germany). Chromatography was conducted at 1–5 MV/min with wash and elution performed using defined buffers. Regeneration was performed with 1 M NaOH, and the column was equilibrated prior to reuse.

#### 2.4.9. Ultradiafiltration 2

Final polishing was performed by crossflow filtration with a 100 kDa MWCO PES or cellulose-based membrane. The product was concentrated and diafiltered with ≥10 diavolumes into formulation buffer (WFI-based). The ultraretentate was maintained at ≤4 mg/mL protein concentration and finally adjusted to 1.5 mg/mL before sterile filtration through a 0.2 µm membrane.

#### 2.4.10. Nanofiltration

For viral clearance, the product was prefiltered through a 0.1 µm filter and passed through a Planova^®^ 20N nanofilter (Asahi Kasei, Tokyo, Japan) operated at 0.70–0.90 bar and room temperature, with a maximum load of 50 kg/m^2^. After filtration, the system was flushed with buffer to minimize product loss. Integrity and leakage testing were performed to confirm filter performance. The final product concentration was adjusted to 1.0 mg/mL using formulation buffer.

### 2.5. Model Viruses for Viral Clearance Studies

#### 2.5.1. Murine Leukaemia Virus (X-MuLV)

X-MuLV is an enveloped, medium-sized (~70–100 nm), single-stranded RNA virus (pNFS Th-1 Xenotropic strain: ATCC VR-1447) with a low resistance to physicochemical inactivation. This class of retrovirus (C-type) is present as an endogenous element in murine hybridomas and in Chinese Hamster Ovary cells (CHO cells). The CHO C-type retroviruses isolated to date have all been defective. X-MuLV is therefore used as a representative non-defective C-type retrovirus. It is obligatory to demonstrate clearance of this class of virus by downstream processing systems used for the purification of biological products from monoclonal CHO-derived recombinant products.

#### 2.5.2. Murine Minute Virus (MMV)

MMV is a non-enveloped, small (~18–25 nm), single-stranded DNA virus (ATCC VR-1346) with a high resistance to physicochemical inactivation. It therefore provides a severe test for the clearance and reduction capacity of the downstream processing system. Incidences of MMV contamination in CHO cell fermenters have heightened the concern with this virus. The tropism of this virus is not fully understood, but it could be a potential contaminant of other biopharmaceuticals produced with a rodent cell line, and is therefore recommended for inclusion in validation studies for products manufactured from CHO or murine derived cell lines. It can also be used as a general model for other parvovirus-like contaminants.

### 2.6. Analytical Methods

#### 2.6.1. Cell Concentration and Viability

Total cell concentration was measured by electronically counting cell nuclei after cell lysis, using a Multisizer™ 4 Coulter Counter (Beckmann Coulter Life Sciences, Brea, CA, USA). Culture viability was assessed through Trypan blue dye exclusion and electronic counting with a Bioprofile Flex2 automated cell culture analyzer (Nova Biomedical, Waltham, MA, USA). Selected samples were manually counted through Trypan blue dye exclusion using a hemocytometer.

#### 2.6.2. Carbohydrates and Amino Acids

Concentrations of glucose, lactate, glutamine, glutamate, and NH_4_^+^ were measured in cell-free supernatants using the BioProfile Flex2 automated cell culture analyzer (Nova Biomedical, Waltham, MA, USA).

#### 2.6.3. Product Concentration

Levels of the product in cell-free supernatants were measured by sandwich ELISA based on capture and detection through antigen binding by mAb mVRC01 [10,17].

#### 2.6.4. Osmolality

The osmolality of culture supernatants in selected samples was determined using the freezing point depression method.

#### 2.6.5. Host Cell Protein Content

The concentration of host cell proteins (HCP) was measured by ELISA with a CHO 360-HCP test kit (BioGenes GmbH, Berlin, Germany).

#### 2.6.6. DNA Content

Host cell DNA in cell-free supernatants was quantified with the PicoGreen^®^ assay (Quant-iT™ PicoGreen™ dsDNA Assay, Thermo Fisher Scientific, Waltham, MA, USA). The assay detects fluorescence (485 nm/528 nm) after dsDNA binding, and quantification is performed using a CHO DNA calibration curve (D552, Cygnus Technologies, Leland, NC, USA).

#### 2.6.7. Antigenicity

The antigenicity of nanoparticles was evaluated by Biolayer interferometry (BLI) analysis. BLI assays were performed on the Octet Red instrument (ForteBio, Sartorius, Menlo Parl, CA, USA) as previously described [9,11]). Anti-human IgG Fc capture biosensors (ForteBio, Sartorius, Göttingen, Germany) were used to immobilize mVRC01, P1B5, 179NC75, and irrelevant mAbs at 20 μg/μL, and baseline interference was measured for 60 s in kinetics buffer (KB: PBS pH7.4, 0.1% bovine serum albumin, 0.02% Tween-20, 0.1% Kathon). Sensors were immersed in wells containing HxB2.WT.Core-C4b at a concentration of 1 μM for 300 s during the association phase, followed by another 300 s in KB during the dissociation phase. All Env-Ab binding measurements were corrected by subtracting the signal obtained from concurrent tracing of the corresponding Env using an irrelevant IgG. Curve fitting was executed using ForteBio’s Data Analysis 9 software.

#### 2.6.8. Electron Microscopy

HxB2.WT.Core C4b samples, produced as described in Section 2.1, Section 2.2, Section 2.3 and Section 2.4 above, were diluted in Tris-buffered saline (50 mM Tris pH 7.4, 150 mM NaCl) to ~0.1 mg/mL and a 3 µL drop was applied to glow-discharged carbon-coated copper mesh grids for 10 s, blotted with Whatman #1 filter paper, and stained with 3 µL of 2% (*w*/*v*) uranyl formate solution for 45 s. Excess stain was blotted with filter paper. Automated data collection was set up using Leginon [18] on either a 120 keV FEI Tecnai Spirit equipped with an FEI Eagle 4K CCD (52,000× magnification; 2.06 Å pixel size), or a 120 keV FEI TF20 equipped with a TVIPS TemCam F416 CMOS (62,000× magnification; 1.68 Å pixel size). Micrographs were saved in the Appion database [19,20] and imported into cryoSPARC v4.6 [21]. Initial particle picking was performed using Blob Picker followed by 2D classification and selection of representative templates for Template Picker. After particle extraction, two rounds of 2D classification were performed with final particle stack numbers of 28,536 (Lot L20 sample) and 16,199 (Lot T206 sample).

#### 2.6.9. Glycosylation Analysis

DeGlyPHER [22] was used to determine site-specific glycan occupancy and processivity on the glycoproteins. Briefly, disulfide bonds on the glycoprotein were reduced and alkylated before digestion with Proteinase K, followed by sequential deglycosylation of glycopeptides with Endo H and then PNGase F in the presence of H_2_^18^O. The peptides were separated on C18 resin using an EASY-nLC 1200 UHPLC (Thermo Fisher Scientific) and nanosprayed into a Q Exactive HF-X mass spectrometer (Thermo Fisher Scientific). Spectra were acquired in data-dependent mode with HCD fragmentation. On the Integrated Proteomics Pipeline (IP2, Integrated Proteomics Applications), extracted tandem mass spectra were searched using ProLuCID against the known glycoprotein sequence within a *Cricetulus griseus* proteome background, with C + 57.02146 Da as a static modification and N + 2.988261 Da (signature for complex glycans), N + 203.079373 Da (signature for high-mannose/hybrid glycans), M + 15.994915 Da, and N-terminal Q–17.026549 Da as variable modifications. These were filtered at the spectrum level up to 1% FDR using DTASelect2 and then quantified with Census2 label-free analysis, applying “match between runs.” GlycoMSQuant was used to compile the final results, aligning potential glycosylation sites to Env of the HXB2 HIV-1 variant.

## 3. Results

### 3.1. Cell Culture Process Evaluation and Optimization with the Stable Pool Cell Line

This initial evaluation aimed to replicate the production protocol provided by DHVI, which used a fixed feeding schedule and fixed processing times. Reproducing this baseline process helped us establish a reference point for later steps optimization.

Building on this, the next goal was to improve process performance by systematically evaluating key variables, including pH and temperature control, feed initiation and timing, and harvest/clarification materials. Each parameter was tested for its effect on cell growth, viability, and product yield.

#### 3.1.1. Effects of pH and Base Addition

Initially, an existing fed-batch protocol was used for a 2 L scale fermentation run. Additional cell culture runs were performed in sets of three parallel bioreactors to explore different conditions during cultivation, such as the effect of pH control through base addition at various pH levels, and to gather information about viability progression in the later process phase. Additionally, two bioreactor runs at an 8–10 L scale were carried out to produce more material for downstream processing (DSP) development and to evaluate the impact of using different bioreactors. An overview of these experiments, along with benchmark data, is provided in Table 1. Bioreactors F977 to F979 were operated in parallel, each starting from the same inoculum culture. F981 was initiated from the same inoculum culture as the small-scale runs F982–F984, while F985 was completed independently with a separate inoculum culture.

Growth and Viability Trends: The maximum cell concentrations across all runs ranged from 8.7 to 13.2 × 10^6^ cells/mL. Runs with base addition for pH control (F978, F979, and F984) showed slightly reduced growth compared to other runs, but there was no noticeable effect on viability progression. Extending cultures beyond the 80% viability threshold provided insights into the robustness of the cell line, revealing a relatively slow decline in viability across all processes.

Product Formation and Yield Variation: Differences in product formation were observed among the different runs. In the first series of cell culture runs (F977–F979), the runs with base addition (F978 and F979) resulted in product yields of approximately 40–50 mg/L when harvested at 80% viability, whereas the run without base addition (F977) yielded 81 mg/L at 87% viability. The second cell culture series (F982–F984) reinforced this observation. While F984 (with base addition) remained comparable to runs F978 and F979, runs F982 and F983 outperformed all previous runs, demonstrating the potential for product yields exceeding 100 mg/L, with some reaching up to 200 mg/L.

Metabolic Shifts and pH Control Effects: A clear link was found between pH control through base addition and lactate metabolism. In processes without base addition (F977, F982, and F983), cells switched to consuming lactate after reaching peak concentrations of about 2 g/L between days 4 and 6. Conversely, processes with base addition for pH adjustment (F978, F979) exhibited continuous lactate buildup throughout, whereas a reduced base addition strategy (F984) resulted in lactate levels stabilizing at around 3 g/L.

Glucose Utilization and Nutrient Dynamics: Since the fed-batch protocol follows a fixed feeding schedule, glucose levels during the process depend on cell culture performance. An overfeeding event was observed on day 4 of the process F983, but overall, glucose concentrations between 2 and 6 g/L were within an acceptable range and did not negatively impact process performance. No limitations in cell-specific glucose levels were observed in any of the processes. Trends for glutamine and ammonium concentrations were similar across all processes, with minor differences. Cultures maintained at lower pH levels appeared to produce more energy from glutamine, resulting in higher ammonia levels during fed-batch cultivation. However, ammonia concentrations exceeding 5 mmol/L NH_4_^+^ were only seen during the late phases of the process when viability was already declining and did not adversely affect the cultures.

#### 3.1.2. Effect of Temperature

A series of temperature-shift experiments was conducted using the standard feeding regimen and a fixed pH setpoint of 6.80 to evaluate the effect of reduced temperatures on growth and productivity. Temperature shifts to 33 °C, 34 °C, and 36 °C were applied during the late exponential phase. The goal was to determine whether lower temperatures could sustain high product yields while decreasing by-product formation and oxygen consumption, potentially facilitating process scale-up. Table 2 summarizes the experimental conditions and results. Small-scale (2 L) runs (F982 020, F983 020, and F984 020) were inoculated from the same culture, along with an 8 L control run (F985) at 37 °C. Cell growth and productivity declined slightly in the temperature-shifted cultures compared to the control and previous 37 °C processes. Lower temperatures during fed-batch resulted in acceptable yields, but reducing the temperature to 33 °C did not significantly improve by-product formation or viability progression. Further results are not discussed, as temperature reduction was not a viable strategy for enhancing process performance.

### 3.2. Subcloning of HxB2.WT.Core-C4b Stable Pool Cell Line and Selection of a Lead Clone

The goal was to establish a clonally derived CHO cell line from a previously generated HxB2.WT.Core-C4b stable pool for creating a GMP-compliant master cell bank (MCB). Subcloning, evaluation, and selection focused on stable growth, productivity, and product quality.

Subclone Generation and Evaluation: Single-cell subcloning was carried out through limiting dilution into 384-well plates, with clonality confirmed using the Cell Metric imaging system. Clones exhibiting strong growth were expanded to 96-well plates. At this stage, supernatants were analyzed by ELISA for product concentration, and confluency data from the Cell Metric system were used to identify the most promising clones. Initially, sixty monoclonal subclones were expanded, and from these, 25 were selected based on expression and growth performance for further testing in 50 mL culture tubes.

Selection of Top Subclones: Routine cultivation data (growth, viability, and productivity) from tube cultures were used to narrow down the panel to ten high-performing subclones. These were adapted to 125 mL shaker flasks and underwent further evaluation, including long-term culture stability, product concentration by ELISA, and fed-batch performance. Research cell banks (RCBs) were created for all ten subclones through cryopreservation in liquid nitrogen.

Fed-Batch Cultivation and Lead Clone Selection: The ten subclones were tested in fed-batch shake flask experiments, where daily monitoring of cell growth, viability, product concentration, and metabolite profiles (glucose, glutamine, lactate, and ammonium) was conducted. All subclones exhibited acceptable growth and viability, but their performance varied in terms of productivity and product quality. A follow-up round of fed-batch testing with the three best-performing clones confirmed the similarity of their growth traits while revealing differences in productivity and glycoprotein quality attributes.

Based on an integrated evaluation of culture stability, productivity, and product quality, clone 4E22 was selected as the leading candidate. This subclone was used to establish the final GMP-compliant master cell bank (CHO/HXB2C/4E22/MCB) for subsequent process verification and cGMP production runs.

### 3.3. Cell Culture Process Evaluation After Subcloning

Clone Evaluation and Feed Adaptation

Cell clones derived from the HxB2.WT.Core-C4b MCB was first tested in shake flask cultures, followed by bioreactor experiments for further evaluation. All bioreactors were inoculated simultaneously, except for F988 (clone 4E22), which included a fill-up step to increase the culture volume for F986 and F987. Clone 4E22 was also used to test fed-batch reproducibility at a different scale and to examine the effects of a glucose-enriched feed solution. Based on glucose consumption data, the feed for F987 was enriched to 120 g/L glucose to keep levels around 3 g/L throughout the process. Other runs followed the standard feeding protocol. The new pH control method, utilizing CO_2_ regulation at the upper deadband and limiting base addition until a pH threshold of 6.65 was reached, was applied to all bioreactors, which were now operated with light protection. Table 3 summarizes the experimental conditions and results. To standardize harvest conditions, all cultures were harvested with viabilities above 80%, and the process duration was limited to 12 days, compared to previous runs that lasted 13–14 days.

Growth and Viability: Maximum cell densities across all scales and clones reached about 12 × 10^6^ cells/mL, with clone 5C9 showing slightly better late-stage growth. Clone 4E22 had higher viability than clones 9K21 and 5C9, despite all meeting the harvest criterion on the same process day. Differences observed in viable cell progression were relatively minor.

**Figure 2:**
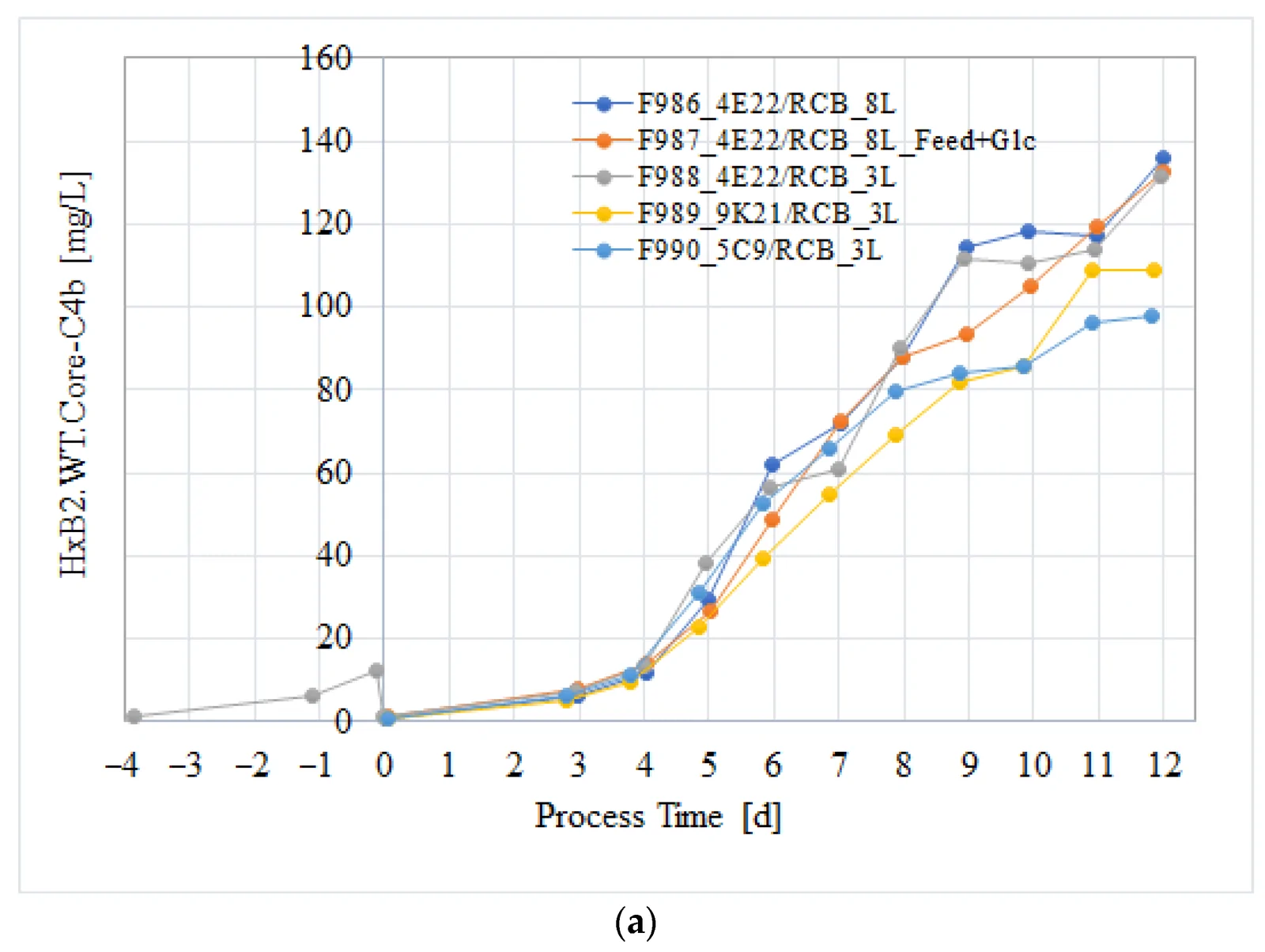

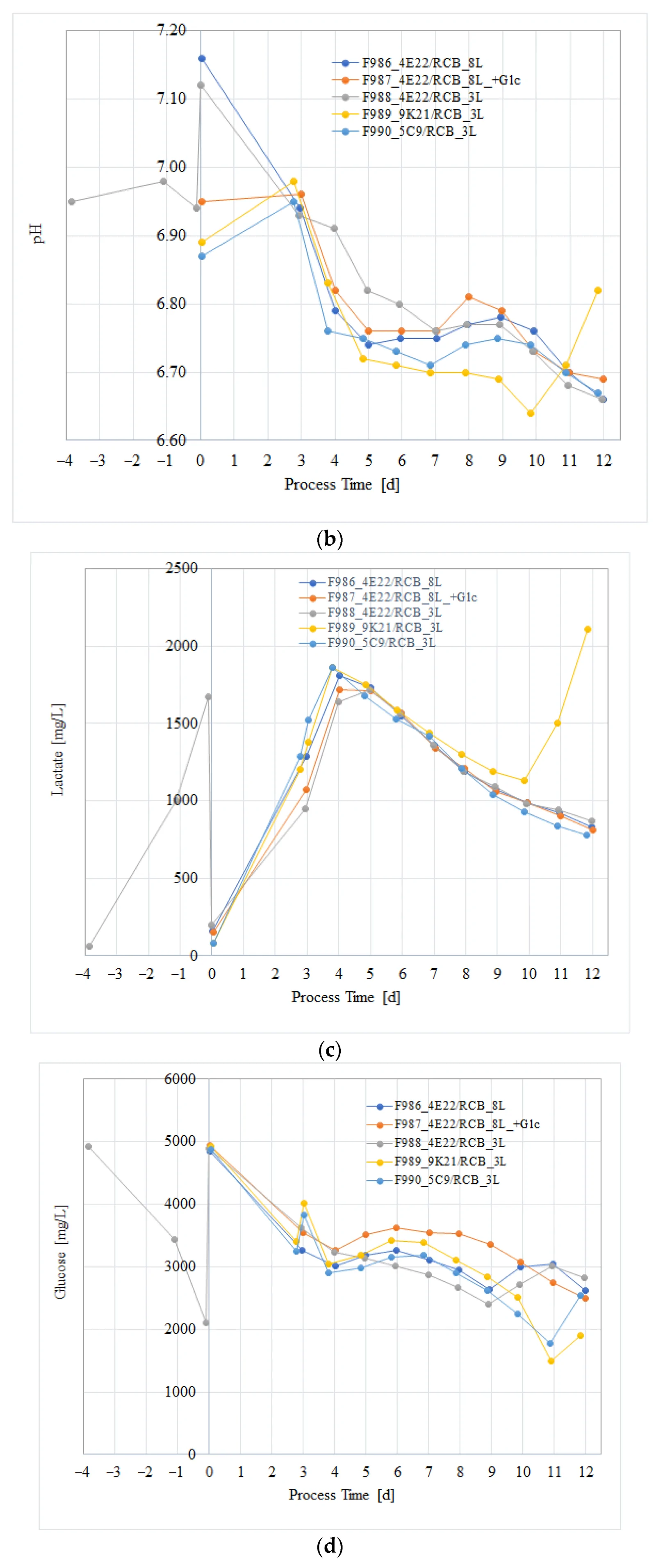

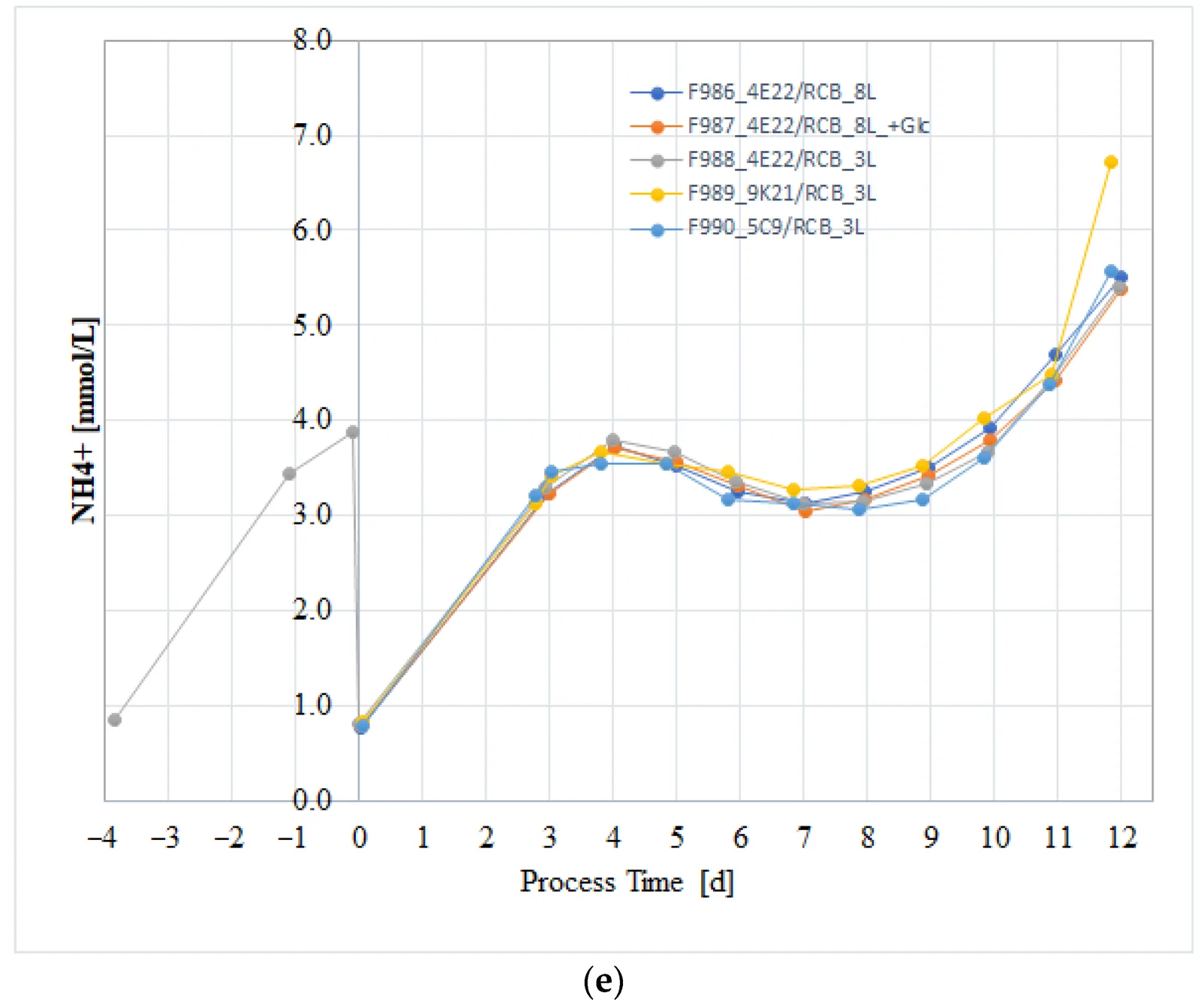
Fed batch performance of the top three subclones 4E22, 9K21, and 5C9, with 4E22 cultivated in three modes: 3 L fill-up simulation (F988), 8 L standard fed-batch (F986), and 8 L Glc-enriched feed (F987). (**a**) Titer, (**b**) pH levels, (**c**) lactate concentration, (**d**) glucose levels, and (**e**) ammonia levels.

Productivity: Clone 4E22 showed higher titers than all other evaluated clones, surpassing 130 mg/L, while clones 9K21 and 5C9 reached around 100 mg/L. Productivity increased after day 4 in all 4E22 runs, while clones 9K21 and 5C9 showed reduced accumulation on the final process day (Figure 2a).

pH and Lactate: pH levels were consistent across runs, except for F989 (clone 9K21), where pH fell below 6.65 on day 10, prompting base addition and causing lactate to rise to 2110 mg/L. Other cultures kept pH above 6.65 throughout (Figure 2b,c).

Glucose Utilization: Glucose levels stayed stable across standard-fed cultures until day 9. To prevent depletion, glucose was added to F986, F988 (clone 4E22), and F989, F990 (clones 9K21 and 5C9) on days 9 and 10. Clones 9K21 and 5C9 used more glucose in the late phase, dropping below 2 g/L by day 10. F987 (glucose-enriched feed) maintained levels above 3 g/L early on and above 2.5 g/L until harvest (Figure 2d).

Ammonium: All cultures showed similar trends, except F989 (clone 9K21), where ammonium buildup increased sharply on the last day, coinciding with lactate accumulation (Figure 2e).

### 3.4. Process Verification of the CHO/HxB2C Production MCB Clone

To verify the performance and reproducibility of the CHO/HxB2C/4E22/MCB production clone under controlled bioreactor conditions, three parallel fed-batch cultivations were performed. Two 10 L runs (F991 and F992) used an identical inoculum culture, while a third 10 L run (F994) involved an independently prepared seed train. For F994, the culture was harvested in two fractions at days 12 and 14 to evaluate the effect of extended process duration. Table 4 summarizes key process parameters and results. Harvested material from all runs was used for downstream process verification.

Cell Growth and Viability: All bioreactor runs exhibited strong cell growth, with peak viable cell densities ranging from 12.4 to 13.8 × 10^6^ cells/mL. Viability stayed high throughout cultivation, with a gradual decrease seen in later stages. Runs F991 and F992 were stopped on day 11 to keep viability above 80%, aligning with typical harvest standards for upstream process consistency. Meanwhile, F994 was intentionally extended, and both harvest fractions (days 12 and 14) maintained viabilities above 70%, demonstrating the clone’s robustness and the process’s capacity for longer operation without harming culture health.

**Figure 3:**
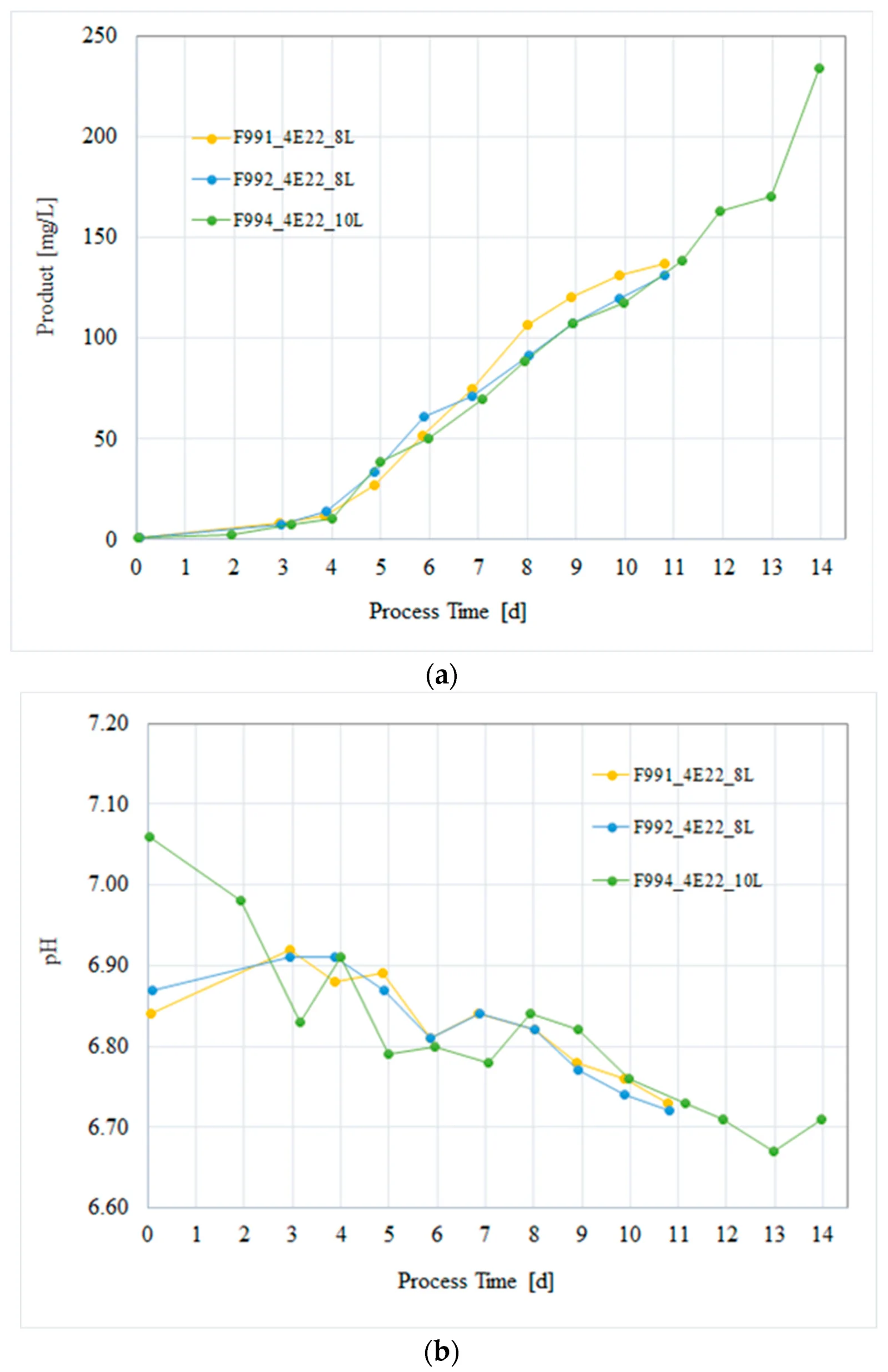

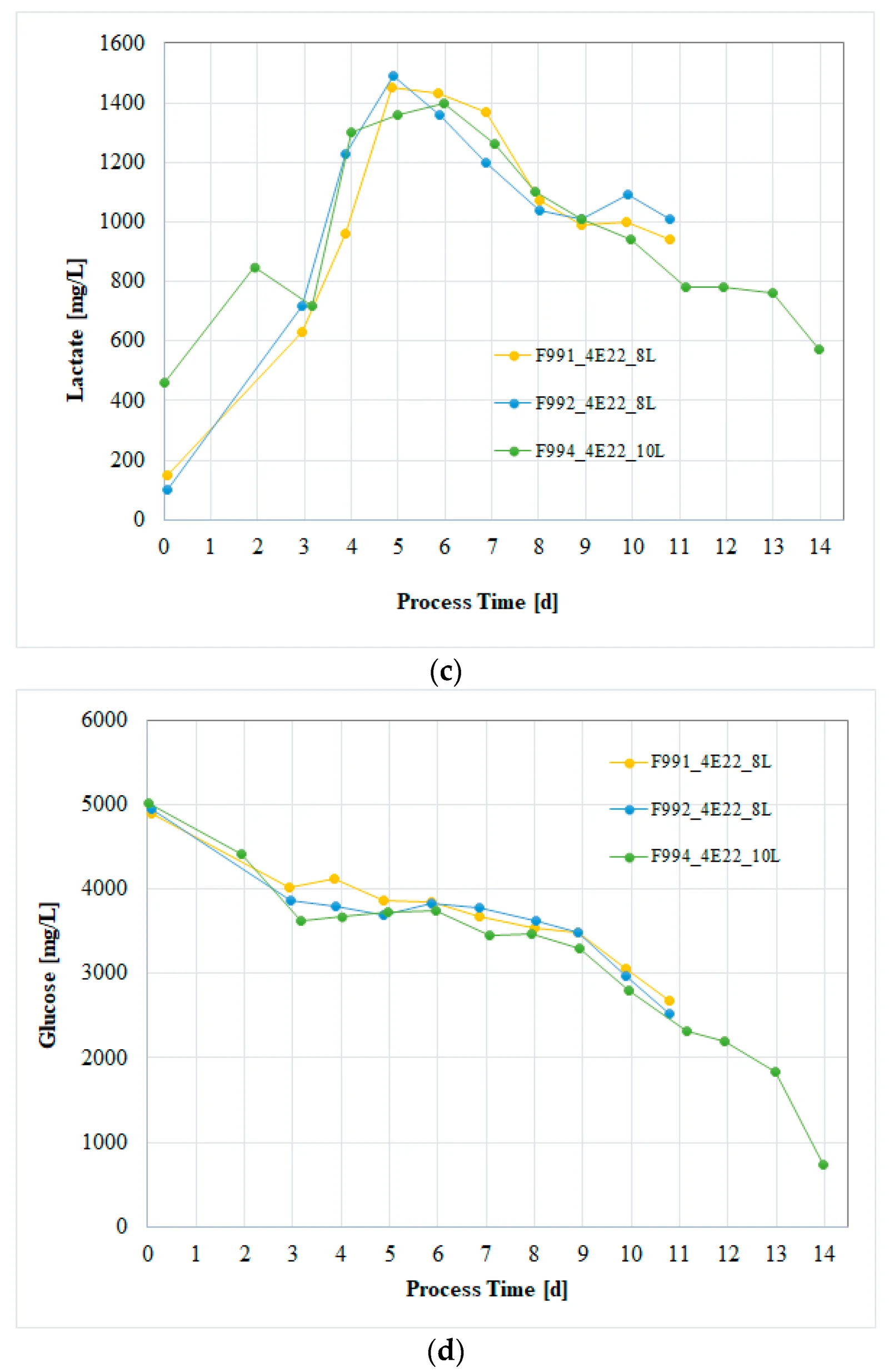
Fed batch performance of the top clone MCBCHO/HXB2C/4E22/MCB cell line at small scale. (**a**) Product concentration, (**b**) pH level, (**c**) lactate levels, and (**d**) glucose. F994 was cultivated for an extended process with harvests at 11 and 14 days.

Protein Productivity: All runs produced product concentrations over 130 mg/L by day 11, confirming consistent clone expression under the established feeding and control strategy (Figure 3a). Notably, F994 showed improved productivity, with projected titers reaching up to 250 mg/L by day 14. This suggests that a slight extension of the cultivation period could increase volumetric productivity. Although F991 showed slightly higher titers during the feeding phase, likely due to better growth kinetics, these differences remained within typical process variation.

Process Metabolites: The pH and lactate profiles were consistent across all runs, maintaining stability without the need for base addition (Figure 3b). Glucose levels remained within control limits throughout, supported by the glucose-enriched feed (Figure 3d). No bolus glucose additions were required. However, in extended runs such as F994, nutrient modeling indicated that supplemental glucose or a reformulated feed may be necessary during the final 48 h to maintain carbon availability. Glutamine and ammonium concentrations followed expected trends across all runs, with minor deviations observed in F994. These were attributed to the detection limits of the metabolite analyzer rather than true biological variation. Ammonium accumulation remained within non-inhibitory ranges, and no adverse effects on growth or productivity were observed.

Implications for cGMP Process Scaling: The reproducibility of cell growth, viability, and productivity across replicate runs, along with stable metabolite profiles, confirms the robustness of the upstream process for the CHO/HxB2C clone. Additionally, the extended culture in F994 demonstrates the feasibility of process intensification, providing an opportunity for yield improvement during scale-up. These findings support the selection of this upstream platform for cGMP manufacturing and supply representative material for downstream process characterization and validation.

### 3.5. GMP Manufacturing Process and Process Controls

#### 3.5.1. Upstream Process: CHO Cell-Based Bioreactor Production

A flow diagram of the cGMP-compliant manufacturing process for HxB2.WT.Core-C4b immunogen is shown in Figure 1. The process includes upstream cell culture operations, downstream purification steps, and final formulation of the drug substance. It begins with thawing a vial from the CHO/HxB2C MCB. Cells are expanded in chemically defined, serum-free media under strictly controlled conditions. The seed train involves sequential sub-cultivation steps to reach high cell densities. Once the culture attains approximately 1.4 × 10^10^ viable cells, it is used to inoculate a 500 L bioreactor (Stainless Steel) operated under cGMP conditions. The bioreactor follows a structured inoculum, batch, and fed-batch strategy. Key process parameters—temperature (set to 37 °C), pH (set to 7.0), and dissolved oxygen (pO_2_ set to 40%)—are continuously monitored and digitally controlled. A nutrient feeding regimen maintains glucose levels and supports high cell viability. The culture is harvested at around 80% viability, corresponding to a cell density of approximately 1.2 × 10^7^ cells/mL.

**Figure 4:**
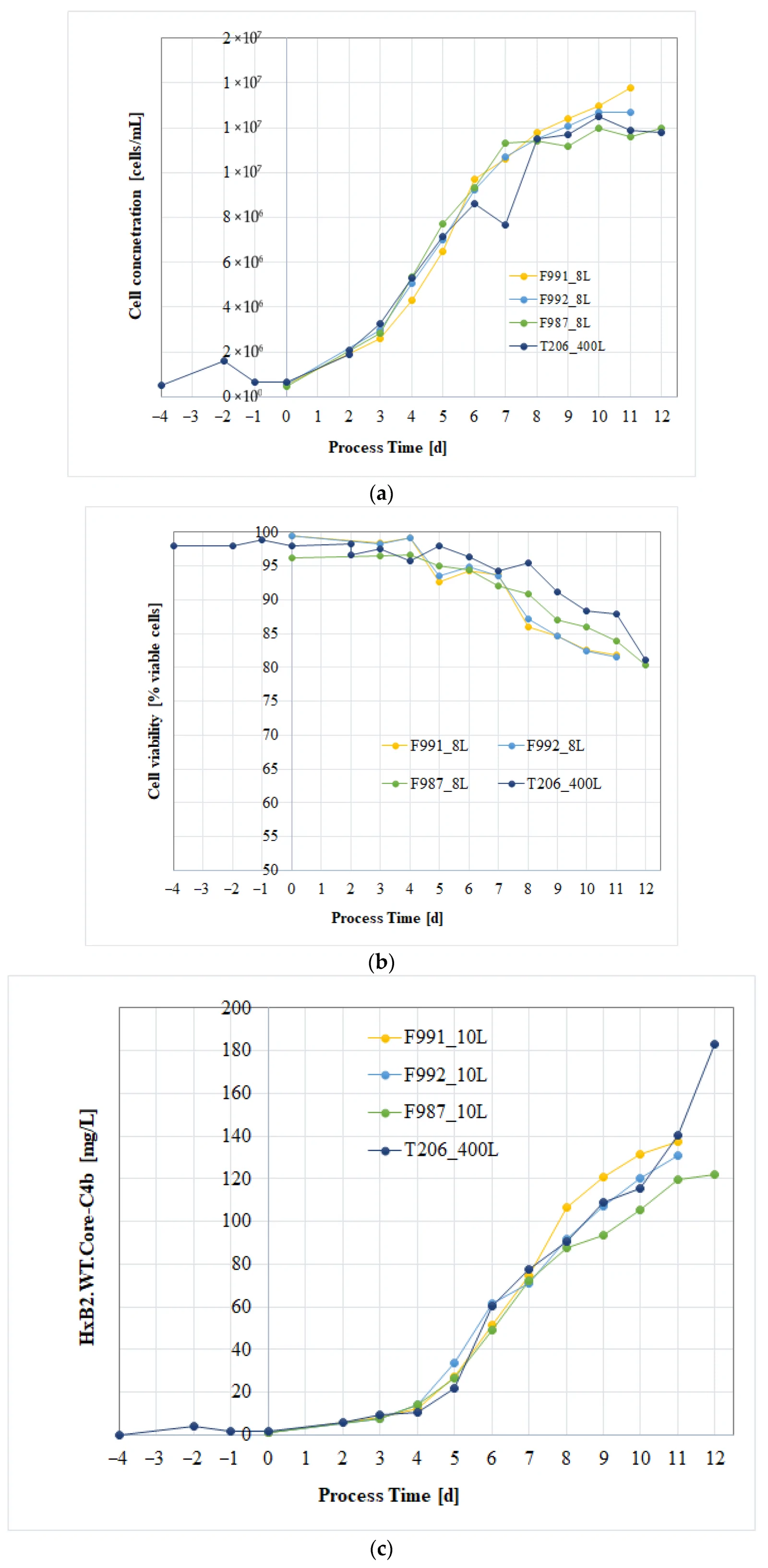
Process performance for confirmation runs (10 L) and full-scale (400 L) production.

The full-scale GMP production run was successfully executed, and the corresponding cell culture performance data are presented in Figure 4a–c. Figure 4a illustrates viable cell density and viability profiles for the process, with direct comparison to representative small-scale runs. The overlay of small- and large-scale data demonstrates that the scale-up was consistent and reproducible, with comparable growth kinetics and viability trends across scales. In addition, the drug substance release testing results from both the small-scale confirmation runs and the full-scale GMP batch are presented in Table 5. All data, including that from the large-scale GMP batch, met the predefined acceptance criteria. Together, these results confirm the robustness of both the upstream cell culture process and the downstream purification process, underscoring the suitability of the platform for GMP manufacturing.

#### 3.5.2. Purification and Process Yield

The downstream purification of the batch T206 harvest involved a multi-step process aimed at gradually removing impurities and enriching the target protein to achieve drug substance quality. Initially, the clarified harvest underwent ultrafiltration/diafiltration (UDF1) and detergent viral inactivation (DVI), with ELISA monitoring to verify target preservation. This was followed by anion-exchange chromatography (AEX1), then hydrophobic interaction chromatography (HAC), and mixed-mode chromatography (CC400) to reduce host-cell proteins and process-related impurities. A polishing cation-exchange step (CEX) was implemented to enhance purity before a second anion-exchange step (membrane adsorber) (AEX2). Subsequent ultrafiltration/diafiltration (UDF2) and nanofiltration (NF) facilitated concentration, buffer exchange, and viral clearance. The process concluded with the final drug substance production, monitored throughout by UV absorbance (A280 nm) for protein recovery.

**Figure 5:**
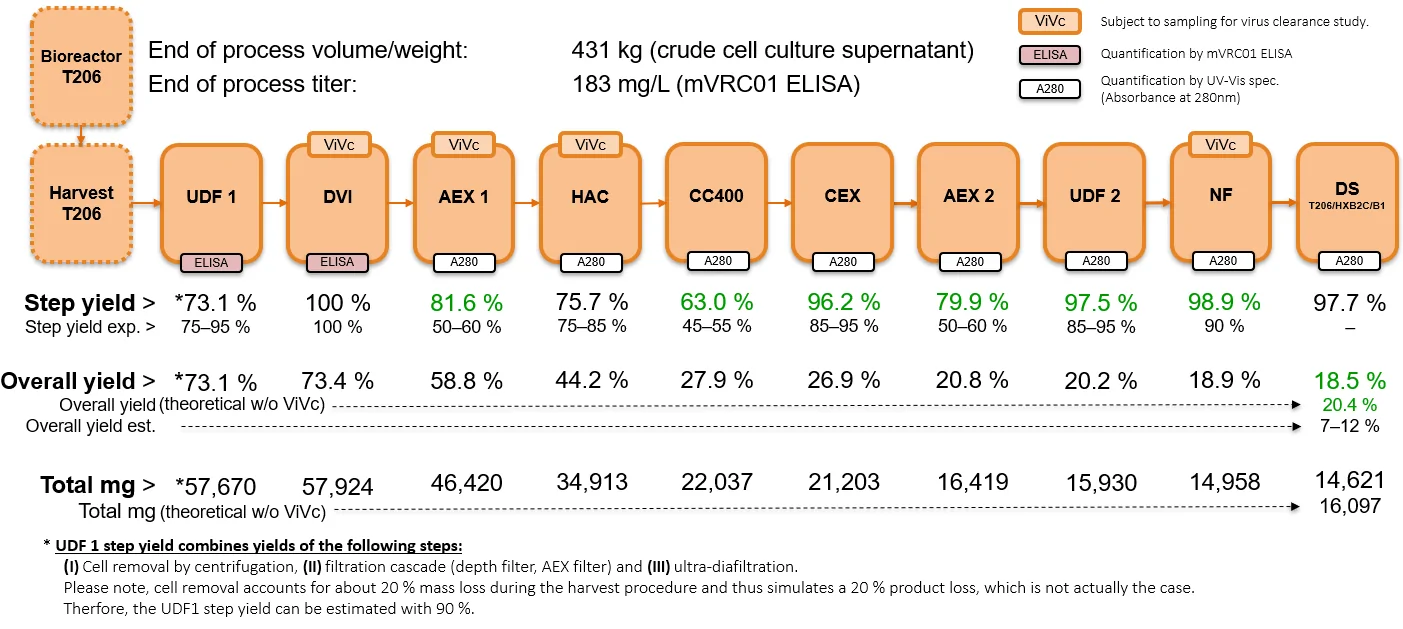
Yield overview (step yields and overall process yields) of the cGMP HxB2.WT.Core-C4b downstream purification sequence.

Yield analysis across steps (Figure 5) revealed variable purification efficiencies, with the most significant product loss occurring at HAC (63.0% yield) and AEX2 (79.9% yield), both of which are critical for high-stringency impurity removal. Conversely, intermediate steps such as AEX1 (81.6%), CEX (96.2%), UDF2 (97.5%), and NF (98.9%) demonstrated high recoveries, reflecting effective control with minimal nonspecific loss. The overall drug substance yield was 18.5%, aligning closely with the historical average of 20.4%. Although overall process efficiency remains comparable, the data suggest HAC and AEX2 as potential yield bottlenecks. These insights emphasize the importance of balancing impurity removal with product recovery, thus highlighting opportunities to optimize these steps and boost overall yield without compromising quality.

Individual step yields are shown as percentage yields relative to the product fraction from the previous step. The expected step yield refers to the minimum and maximum yields obtained during the development and optimization phases to provide context for individual cGMP step yields. Yields highlighted in green indicate steps where the yields exceeded the expected yields during development. The overall yield reflects the cumulative process yield after each corresponding step. Since overall yields are influenced by sampling for the virus clearance and validation studies, corrected overall yields (expressed as percentages or absolute product amounts in mg) are also provided as “theoretical w/o ViVc” values.

### 3.6. Viral Clearance Evaluation

A viral clearance study was conducted to assess the effectiveness of the downstream purification process in inactivating and removing potential viral contaminants from the recombinant HxB2.WT.Core-C4b immunogen. The study was performed by ViruSure GmbH (Austria) under contract, following international regulatory guidance, including ICH Q5A (R2) and EMA guidelines on viral safety evaluation of biotechnological investigational medicinal products. A virus spiking method was used to measure viral reduction at specific downstream processing (DSP) steps. Model viruses, such as the enveloped retrovirus XMuLV and the non-enveloped parvovirus MMV, were intentionally added to the process intermediate material. Viral clearance was then assessed across four distinct unit operations within the production process.

The following DSP steps were evaluated:

Detergent Virodex TXR-1treatment (effective): This step effectively inactivated enveloped viruses through disruption of the viral envelope.

Anion exchange chromatography (partially effective): For anion exchange chromatography, Sartobind STIC PA membrane adsorber capsule (Sartorius, Göttingen, Germany) was used. The HxB2.WT.Core-C4b protein was recovered in the eluate fraction. Viral reduction was mediated by physicochemical partitioning; no chemical inactivation was expected based on buffer conditions.

Hydroxyapatite chromatography (non-effective): Similar to AEX, virus removal was driven by partitioning, with limited contribution to inactivation.

Nanofiltration (effective): A Planova 20N virus filter was used to retain viruses by size exclusion, providing a robust virus removal mechanism.

The combined viral clearance data demonstrated strong process performance (Table 6). For the enveloped model virus X-MuLV, the overall logarithmic reduction factor (LRF) was estimated at approximately 13.66, indicating a high safety margin. For the more resistant non-enveloped MMV, the overall LRF was around 7.65, which aligns with the expected lower susceptibility of non-enveloped viruses to inactivation. Notably, detergent treatment was not tested on MMV due to its resistance to such methods.

An estimate of residual viral particles per clinical dose was calculated based on data from virus-like particles in the unprocessed harvest. For a single 300 µg dose of immunogen, the viral load was less than 1.3 × 10^−5^ particles, or fewer than 4.6 × 10^−3^ particles per mg of protein. These figures indicate a viral safety factor greater than 10^5^, corresponding to over 75,000 doses with no detectable particles.

Collectively, the viral clearance study confirms that the downstream process offers strong and multifaceted viral reduction capabilities. The combination of chemical inactivation and physical removal steps effectively reduces the risk of viral contamination, ensuring product safety for clinical evaluation at this stage of development. Additional results and methodology are detailed in a separate publication [23].

### 3.7. Product Characterization

#### 3.7.1. *N*-Glycosylation Profiling and Impact of Manufacturing Scale on Glycan Occupancy

**Figure 6:**
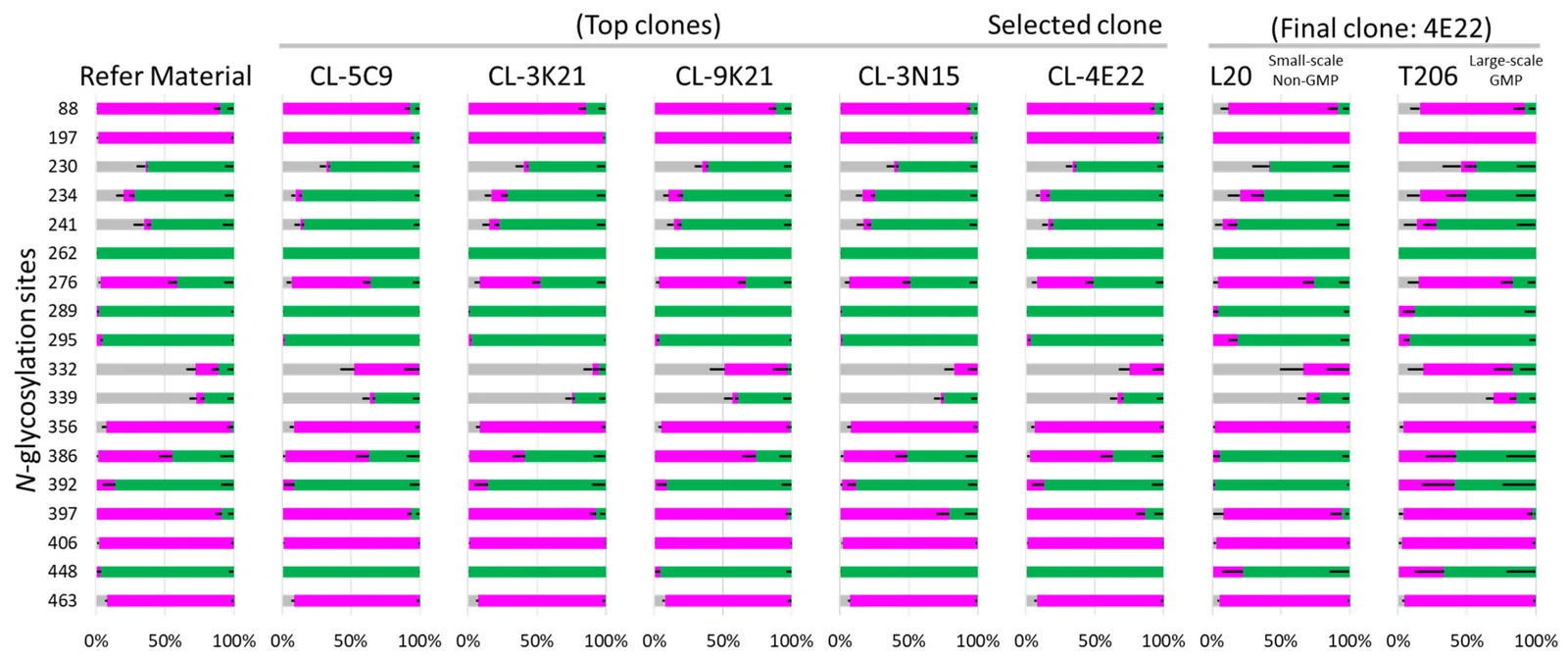
Site-specific *N*-glycosylation analysis. Bar graphs representing proportion of glycosylation and *N*-glycan types at each of the 18 *N*-glycosylation sites on the analyzed immunogen (HxB2.WT.Core-C4b) and its clones expressed in CHO cells. Color code represents proportion of glycosylation state: gray (unoccupancy), magenta (complex glycans), and green (high mannose or hybrid glycans). Error bars represent negative standard error of mean (SEM).

A comprehensive site-specific *N*-glycosylation analysis (DeGlyPHER) was performed on the HxB2.WT.Core-C4b immunogen to evaluate consistency in glycosylation across clones and manufacturing scales, an essential attribute for viral glycoprotein immunogens [22]. Glycosylation was assessed at 18 predefined N-linked glycosylation sites across five clones during the cell line selection stage and further characterized for the selected clone (CL-4E22) at both small-scale (10 L, L20) and large-scale (500 L, T206) production levels (Figure 6).

Across all five clones evaluated during clone selection, glycan profiles were highly consistent in terms of site occupancy and glycan type distribution, and any site-specific difference observed were statistically insignificant (Benjamini–Hochberg adjusted *p*-value < 0.05). The N276 site, known for its importance in modulating epitope accessibility and bNAb targeting [24], demonstrated over 85% occupancy in all clones, reinforcing its structural importance and reproducible manufacturability.

Clone CL-4E22 was selected based on productivity and growth characteristics profile. In addition, we confirmed that ns-EM and glycosylation data are satisfactory for this selected clone. Upon scale-up, *N*-glycan occupancy and composition remained highly consistent between L20 and T206 lots (Figure 6). The examined immunogens/clones exhibited similar site-specific distribution of complex (magenta) and high-mannose/hybrid (green) glycans, with minimal unoccupied sites (gray), supporting structural homogeneity. Interestingly, at the N332 site—critical for bNAb binding [25]-glycan occupancy was usually low but variable between some clones; however, the differences were not statistically significant (Benjamini–Hochberg adjusted *p*-value < 0.05) to infer any deviation in product quality.

These results demonstrate that the glycosylation pattern of the HxB2.WT.Core-C4b nanoparticle is robust to scale-up, with no significant changes in glycan composition or occupancy detected between small- and large-scale batches. The data support the consistency in the quality of this immunogen across manufacturing stages, a critical requirement for advancing into clinical development.

#### 3.7.2. Electron Microscopy Characterization of Nanoparticle Assembly

**Figure 7:**
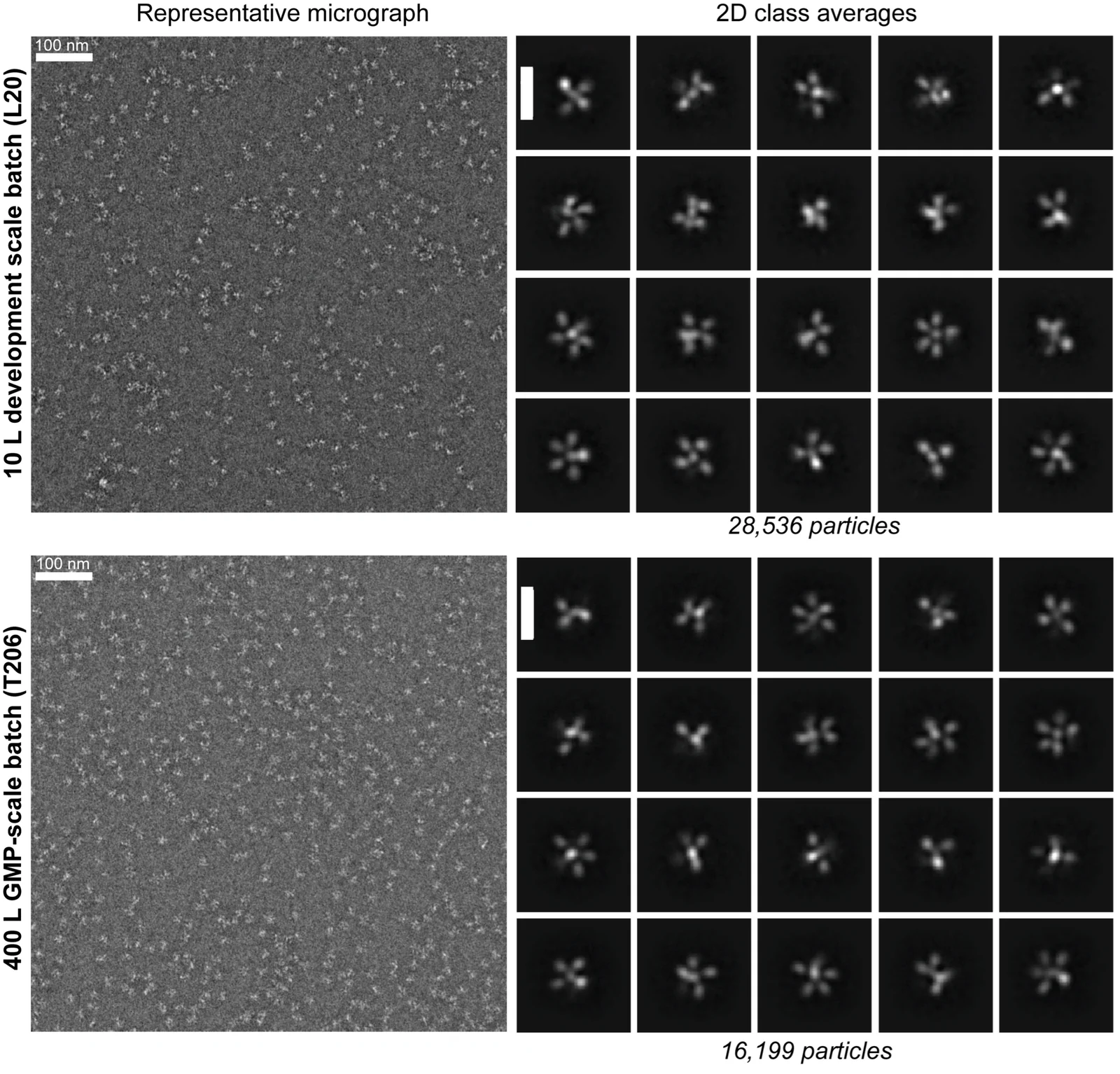
EM images of the material from 10 L and 400 L scale production batches (Clone CL-4E22).

Negative-stain EM imaging of the materials from 10 L scale (Lot L20) and 400 L scale (Lot T206) production batches (Clone CL-4E22). Representative micrographs are shown on the left and 2D class averages on the right. The number of total particles analyzed by 2D classification is labeled for each dataset. The scale bar in 2D class averages represents (Figure 7).

A majority of 2D class averages appeared as a central core density with four or five protruding propeller blade-shaped lobes. This is consistent with C4b-based self-assembling nanoparticles that are expected to form 5-, 6-, or 7-mer assemblies [26]. Due to limitations in 2D views, nanoparticle flexibility and partial occlusion due to stain, the number of visible lobes varied. These findings are consistent with the observed degree of assembly in prior clone selection studies for this construct. Notably, both lots demonstrated a high degree of monodispersity and comparable morphology, with no significant concerns identified by EM alone.

Together, these results support the structural consistency and scalability of the nanoparticle-based immunogen, reinforcing its suitability for advancement under cGMP conditions.

### 3.8. Antigenic Profile by Biolayer Interferometry

**Figure 8:**
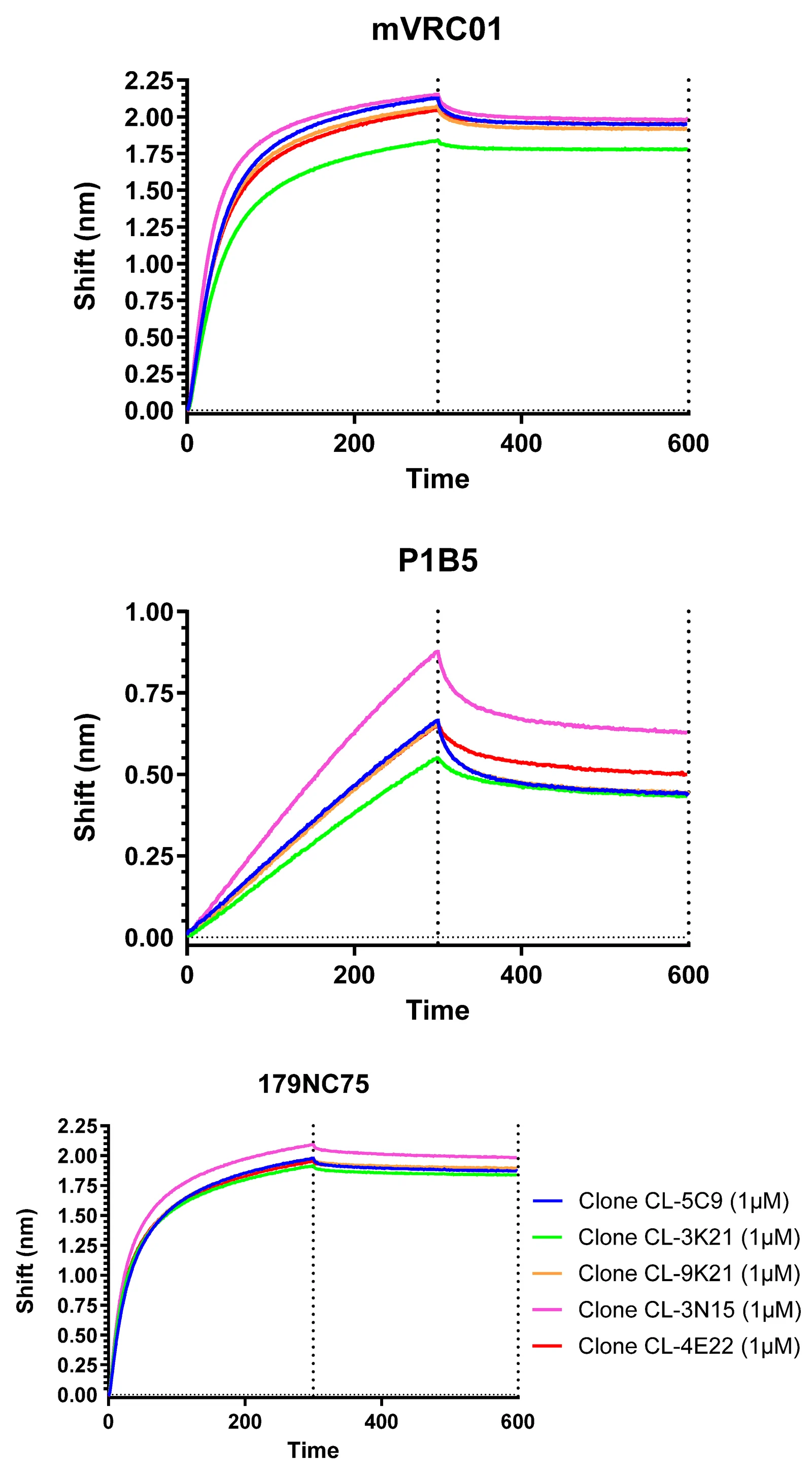

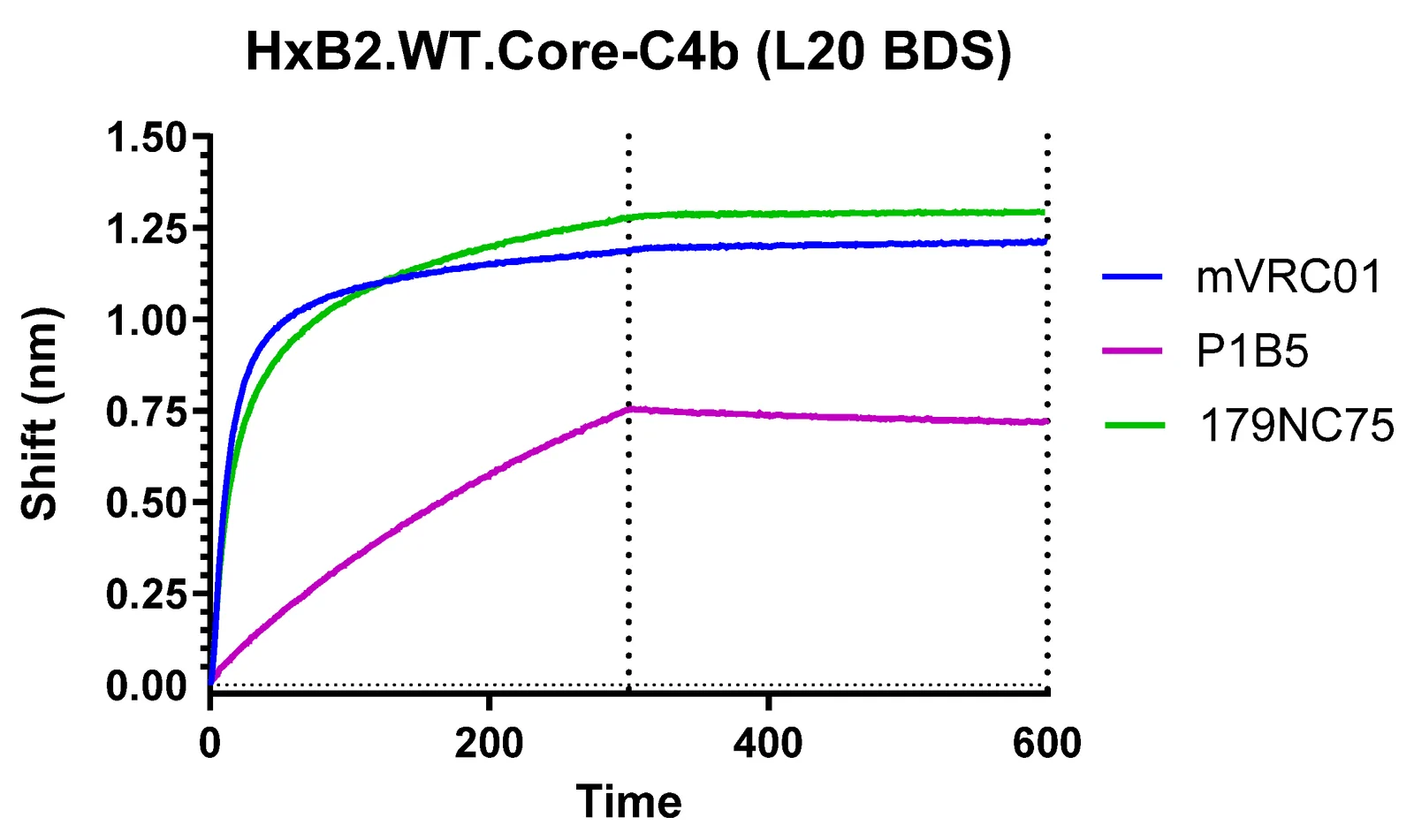
BLI traces of top five clones CL-5C9, CL-3K21, CL-9K21, CL-3N15, CL-4E22, and L20 BDS binding to mVRC01, P1B5, and 179NC75 mAbs.

**Figure 9:**
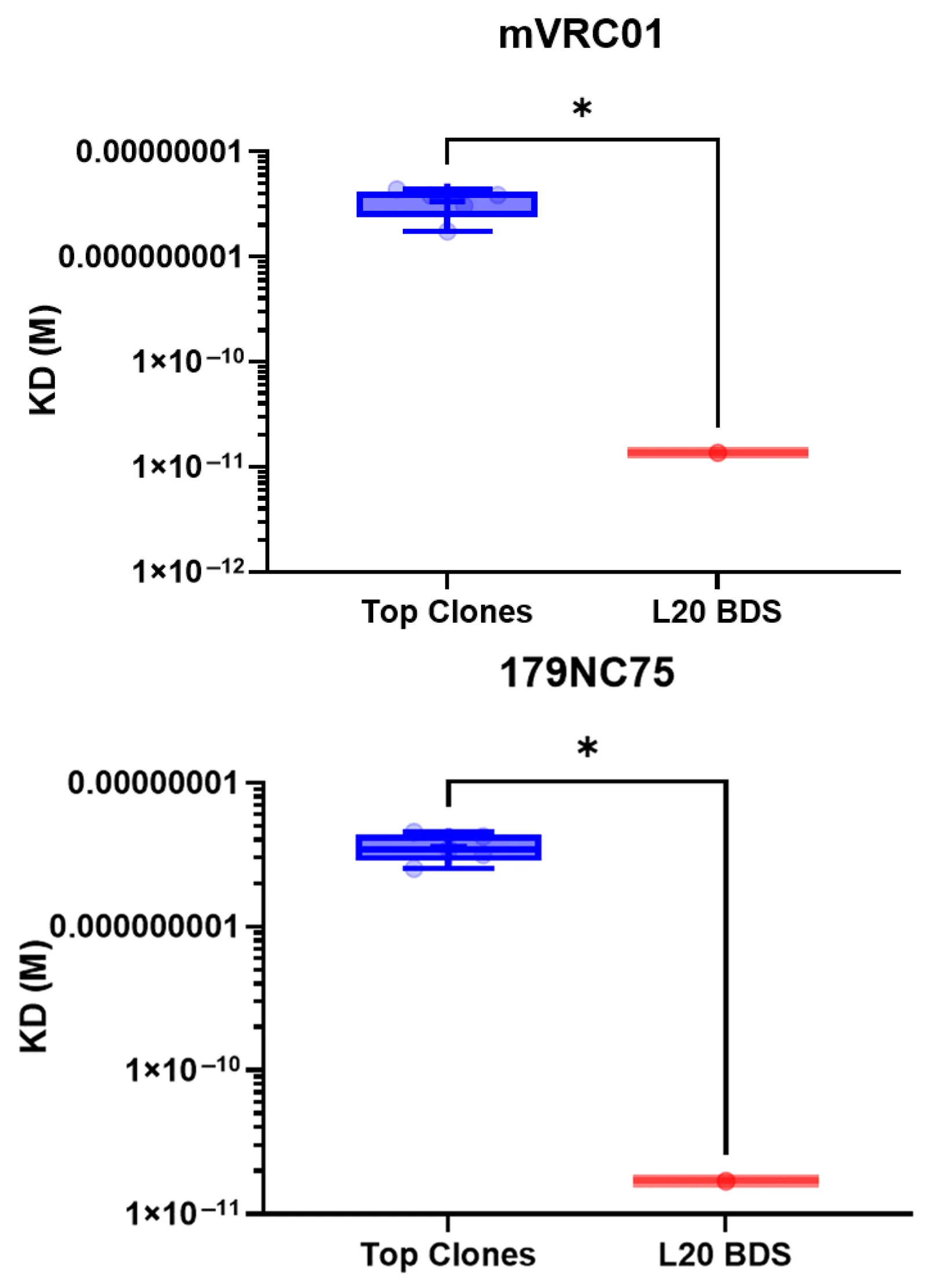

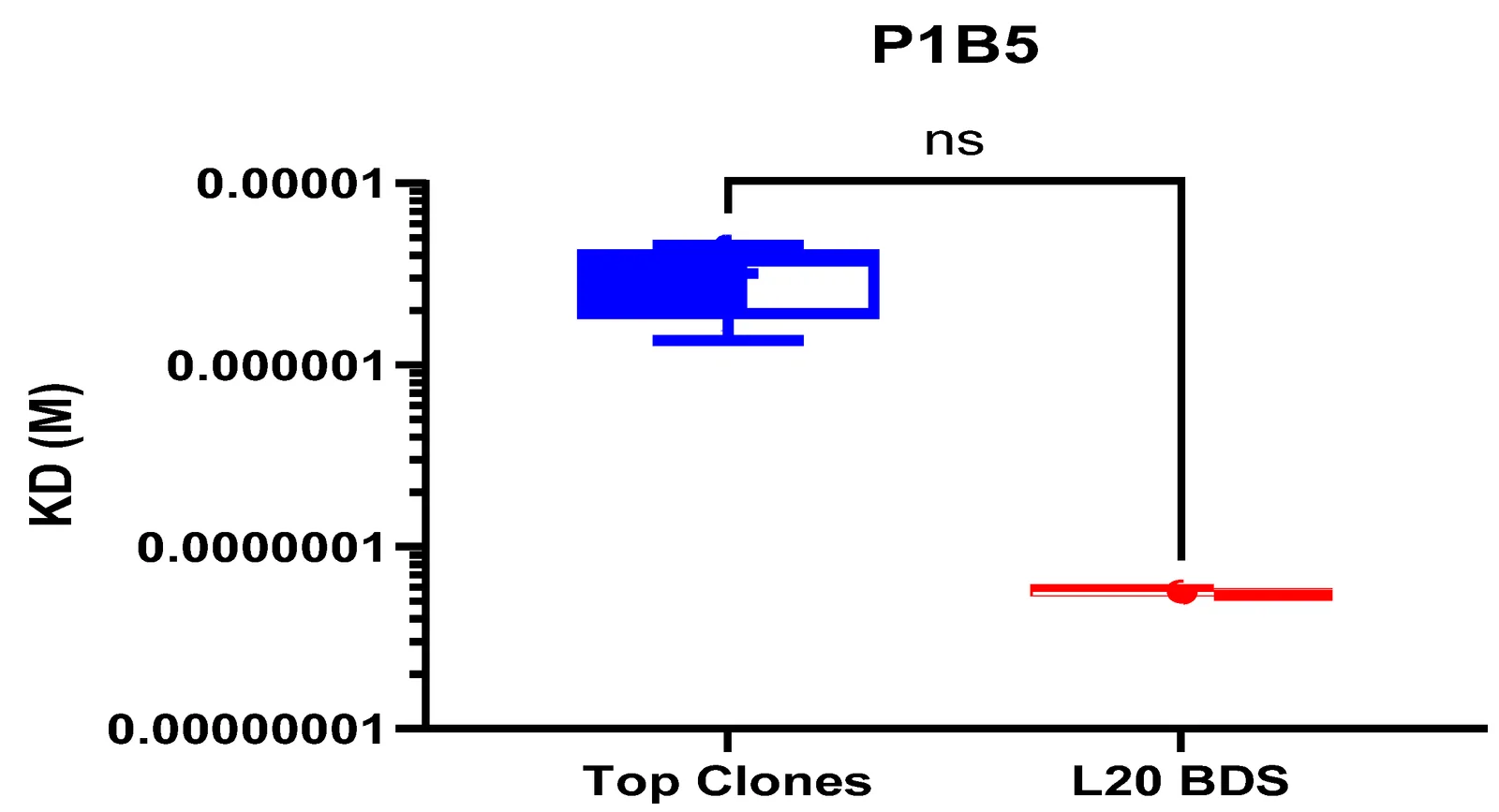
BLI binding affinity (KD) comparison of top clones and L20 BDS against mVRC01, P1B5, and 179NC75 mAbs (*: *p* ≤ 0.05).

Top five clones and confirmation run L20 BDS were evaluated for Env-mAbs binding by biolayer interferometry (BLI). Envs were tested against mVRC01, P1B5 and 179NC75 mAbs [10,17]. BLI assays were performed as previously described [9,11]. Anti-human IgG Fc capture biosensors (ForteBio/Sartorius, Menlo Park, CA, USA) were employed to immobilize mAbs. Sensors were immersed in wells containing Envs during the association phase, followed by kinetic buffer during the dissociation phase. All Env-Ab binding measurements were corrected by subtracting the signal obtained from concurrent tracing of the corresponding Env using an irrelevant IgG. All five clones have similar antigenic profiles with no significant differences across three mAbs binding (Figure 8 and Table 7). BLI analysis of the confirmation run L20 BDS antigenicity demonstrates that, after undergoing five column purification steps, its antigenicity significantly improved, as evidenced by enhanced binding to mVRC01 and 179NC75 mAbs. Binding to P1B5 mAb remained consistent with that of the clone purified through a single size exclusion column step (Figure 9).

## 4. Discussion

### 4.1. Development Runs with the First Cell Line

The cell culture process time needed to reach the harvest criteria was 1 to 2 days shorter than expected, likely because of decreased cellular stress from reducing gas flow through a lower dissolved oxygen (DO) setpoint and maintaining overall low shear forces. Lowering the pH setpoint during the process significantly increased the final product concentration by reducing or eliminating the need for base addition. In contrast, a temperature shift to lower levels did not improve process performance, as cell-specific productivity was negatively affected. However, even at 33 °C, an acceptable yield was maintained. Additional observations showed that adding antifoam was unnecessary, and light protection should be used to maintain process stability. Filtration studies demonstrated that other depth filter materials were suitable, and the introduction of an AEX step after depth filtration was achieved by adding a MAQ absorber (Emphaze, 3 M) step, which effectively reduced impurities at harvest.

Lowering the pH setpoint decreased lactate buildup and reduced the need for base addition, stabilizing osmolality and lowering metabolic stress on the cells. This supports previous findings that mild acidification can shift CHO metabolism from lactate production to more efficient nutrient use, thus enabling higher productivity. Importantly, no changes were observed in nanoparticle assembly or *N*-glycosylation profiles, confirming that product quality attributes stayed stable under these altered conditions. The temperature shift slowed metabolic rates and cell growth, which likely contributed to lower specific productivity despite potential benefits for protein folding and stability seen in other CHO processes. Consistent glycosylation and antigenicity across batches indicate that product quality was not negatively affected by temperature changes.

### 4.2. Clone Selection and the New Production Cell Line

Among the evaluated clones, 4E22 demonstrated superior performance and was selected to produce the new MCB. Process stability was further improved by adjusting the concentrated feeding solution; increasing the glucose concentration to 120 g/L maintained stable glucose levels throughout the fed-batch process, eliminating the need for an additional glucose feed line. After implementing the optimized process protocol, verification runs at different scales were successfully conducted. When harvested at 80%, final product concentrations consistently exceeded 130 mg/L, demonstrating process robustness and reproducibility.

### 4.3. cGMP Production and Process Performance Comparison

The HxB2.WT.Core-C4b downstream process was evaluated across three laboratory-scale batches (L20–L22) and a full-scale GMP run (T206), representing a 40-fold scale-up (Table 5). The process demonstrated consistent product quality across different scales, supporting its robustness and scalability, which are essential in bioprocess development and technology transfer.

General Attributes and Identity: All batches met acceptance criteria for appearance, pH, and osmolality. The pH stayed within the target range (7.4–7.6), and osmolality values were consistent (223–225 mOsmol/kg), indicating well-controlled formulation conditions after purification. Identity was confirmed using ELISA-based antibody binding assays, with all batches showing reactivity to mVRC01, P1B5, and 179NC75, consistent with previous characterization of this immunogen [10]. No loss of antigenic structure was seen upon scale-up.

Potency and Protein Content: Potency was assessed through EC_50_ titration against three monoclonal antibodies. EC_50_ values were consistent across scales, with full-scale results closely matching those from development batches. The total protein concentration measured by UV-Vis spectroscopy remained within the specified range (1.0 ± 0.2 mg/mL) for all tests, indicating stable product yield and concentration control.

Purity Attributes: SEC-HPLC analysis showed minimal aggregation (<0.5%) in small-scale runs and no aggregates in the GMP batch. The main peak area increased from around 99.7% at the developmental stage to 100% at full scale, highlighting improved resolution of product-related species. Residual host cell proteins (HCPs) were well below the 100 ng/mg specification, ranging from 24 to 53 ng/mg, with the full-scale batch slightly higher but still compliant. Residual DNA content, measured by qPCR (Charles River Laboratories, Erkrath, Germany) or Threshold immunoassay (Molecular Devices, San José, CA, USA), stayed below 15 pg/mg across all batches, well below the regulatory limit of 1 ng/mg, in accordance with ICH Q6B guidelines [27]. SDS capillary electrophoresis (SDS-CE) confirmed high product purity in all batches, with main peak intensities ≥ 97%. Impurity profiles were similar between batches, with minimal low- or high-molecular-weight species, suggesting consistent purification performance.

Contaminants and Bioburden: The full-scale GMP batch exhibited low endotoxin levels (<0.16 EU/mg) and no detectable microbial contamination (0 CFU/10 mL), confirming the effectiveness of upstream control and downstream sanitization protocols [28]. Nanoparticle Characterization: Dynamic light scattering analysis showed consistent average particle size (Zavg 18.1–18.5 nm) and low polydispersity indices (PDI ≤ 0.04), indicating a uniform nanoparticle population across all scales. The similarity of these values between development and GMP batches supports the robustness of the particle self-assembly process even during scale-up.

Scalability and Process Robustness: The results verify the reproducibility of critical quality attributes (CQA) across a 40-fold scale-up, demonstrating strong process control and scalability. This consistency is vital for late-stage process validation and regulatory approval in vaccine or biologics development [29]. These findings confirm the platform’s flexibility, with potential for future scale adjustments or technology transfers. These data verify the successful scale-up of the upstream process to 400 L under cGMP conditions, with strong cell growth, sustained viability, and high productivity. The demonstrated scalability and robustness support readiness for clinical material production and build confidence in the platform for future manufacturing campaigns.

## 5. Conclusions

This study details the successful development, scale-up, and analytical characterization of the HxB2.WT.Core-C4b nanoparticle, an HIV booster immunogen for germline-targeting vaccines aiming to elicit anti-CD4 binding site bnAbs. We employed a risk-based, expedited, phased strategy to develop, scale up, and characterize the nanoparticle, which enabled cGMP drug product release within eight months while significantly increasing yield, producing 14.6 g of purified protein to meet clinical material demands. This work directly supported the initiation of a Phase I trial (HVTN 320, NCT06796686), with both small- and 400 L GMP-scale batches demonstrating consistent product quality, epitope integrity, and reproducible glycosylation at 18 sites, including bnAb-associated epitopes. Beyond HIV, the platform provides a model for manufacturing glycosylated nanoparticle vaccines, with future efforts aimed at reducing costs for global access and extending these advances to priming immunogens. Overall, this study establishes a robust, flexible, cGMP-compliant platform that expedites early clinical evaluation of next-generation vaccines designed to elicit broadly neutralizing antibodies.

## Figures and Tables

**Table 1:** Summary of bioreactor runs with the stable pool cell line under various conditions.

Process Code	Test Parameter	Process Parameters DO [%]/T [°C]/pH	Process Duration [d]	Final Viability [%]	Max. Cell Conc. [10^6^ c/mL]	Final Yield [mg/L]
F977 010	No base addition	40/37.0/6.95 *	12	86.7	10.6	81
F978 010	Standard protocol	40/37.0/6.95	13	70.3	9.4	42
F979 010	Extended culture	40/37.0/6.95	14	46.8	10.1	87
F982 010	No base addition	40/37.0/6.95 *	13	64.2	10.6	120
F983 010	No base addition, feed start 1 day later	40/37.0/6.95 *	13	60.3	10.5	161
F984 010	Minimum base addition	40/37.0/6.80	13	78.8	8.74	40
F981 010 (10 L)	Standard protocol	40/37.0/6.95	14	66.0	13.2	117
F985 010 (8 L)	Standard protocol, minimum base addition, including fill-up step	40/37.0/6.80	3 + 13	77.9	12.0	107

* pH control at an initial setpoint of 6.95 with CO_2_ only, which resulted in decreasing pH values during later process time (lower threshold = 6.65).

**Table 2:** Summary of bioreactor runs with the pool cell line at various temperature levels.

Process Code	Test Parameter	Process Parameters DO [%]/T [°C]/pH	Process Duration [d]	Final Viability [%]	Max. Cell Conc. [10^6^ c/mL]	Final Yield [mg/L]
F982 020	Temperature shift to 33 °C	40/37.0 → 33.0/6.80 *	14	74.1	8.97	64
F983 020	Temperature shift to 34 °C	40/37.0 → 34.0/6.80 *	14	72.9	9.96	84
F984 020	Temperature shift to 36 °C	40/37.0 → 36.0/6.80 *	14	76.7	10.6	65
F985 010 (8 L)	Standard protocol, minimum base addition, including fill-up step	40/37.0/6.80 *	3 + 13	77.9	12.0	107

* pH control at an initial setpoint of 6.95 with CO_2_ only, which resulted in decreasing pH values during later process time (lower threshold = 6.65).

**Table 3:** Summary of bioreactor runs using different subclones of ‘HXB2 WT Core -C4B RCB’.

Process Code	Test Parameter	Process Parameters DO [%]/T [°C]/pH	Process Duration [d]	Final Viability [%]	Max. Cell Conc. [10^6^ c/mL]	Final Yield [mg/L]
F986 010 (8 L)	Clone 4E22/RCB	40/37.0/6.80 *	12	81.7	11.9	136
F987 010 (8 L)	Clone 4E22/RCB Enriched feed solution (120 g/L Glc)	40/37.0/6.80 *	12	80.3	12.0	132
F988 010	Clone 4E22/RCB	40/37.0/6.80 *	4 + 12	78.6	11.9	132
F989 010	Clone 9K21/RCB	40/37.0/6.80 *	12	77.3	12.0	109
F990 010	Clone 5C9/RCB	40/37.0/6.80 *	12	79.4	12.6	98

* pH control at an initial setpoint of 6.95 with CO_2_ only, which resulted in decreasing pH values during later process time (lower threshold = 6.65).

**Table 4:** Overview of bioreactor runs with the new production cell line “CHO/HXB2C/4E22/MCB”.

Process Code	Test Parameter	Process Parameters DO [%]/T [°C]/pH	Process Duration [d]	Final Viability [%]	Max. Cell Conc. [10^6^ c/mL]	Final Yield [mg/L]
F991 010	Verification run	40/37.0/6.95 *	11	81.8	13.8	137
F992 010	Verification run	40/37.0/6.95 *	11	81.6	12.7	131
F994 010 (10 L)	Verification run, two different (late) harvest time points	40/37.0/6.95 *	12 (1) 14 (2)	74.9 (1) 70.6 (2)	12.4	163 233

* pH control with CO_2_ only, which resulted in decreasing pH values during later process time (lower setpoint = 6.65).

**Table 5:** Drug substance release testing from confirmation and a full-scale cGMP batch.

Drug Substance Specifications		Results Small Confirmation Runs			GMP Campaign
Test/Analysis	Acceptance Criteria	L20	L21	L22	Drug Substance: T206/HXB2C/B1
General					
Appearance	clear to opalescent, colorless to off-white liquid, essentially free from visible particulates	pass	pass	pass	pass
pH	7.5 ± 0.5	7.5	7.6	7.5	7.4
Osmolality	220 ± 50 mOsmol/kg	224 mOsmol/kg	225 mOsmol/kg	225 mOsmol/kg	223 mOsmol/kg
Identity					
Binding activity (ELISA)	binding to antibodies mVRC01, P1B5, 179NC75 detected	binding to three antibodies detected	binding to three antibodies detected	binding to three antibodies detected	binding to three
					antibodies detected
Potency					
Antibody titration ELISA	report result of the EC50 in ng/mL for antibodies mVRC01, P1B5, 179NC75	mVRC01: 54.3 ng/mL P1B5: 181.9 ng/mL 179NC75: 297.8 ng/mL	mVRC01: 45.7 ng/mL P1B5: 146.4 ng/mL 179NC75: 296.1 ng/mL	mVRC01: 49.6 ng/mL P1B5: 205.3 ng/mL 179NC75: 283.2 ng/mL	mVRC01: 44.2 ng/mL P1B5: 296.4 ng/mL 179NC75: 272.1 ng/mL
Content					
Total protein concentration (UV-Vis 280nm)	1.0 ± 0.2 mg/mL	0.97 mg/mL	1.00 mg/mL	0.95 mg/mL	1.0 mg/mL
Purity					
Purity (SEC HPLC)	<10% Aggregates report result [%] for main peak area	0.34% Aggregates 99.66% Main peak	0.41% Aggregates 99.59% Main peak	0.24% Aggregates 99.76% Main peak	0% Aggregates 100% Main peak
Residual host cell protein (ELISA)	≤100 ng/mg	35 ng/mg (ppm)	29 ng/mg (ppm)	24 ng/mg (ppm)	53 ng/mg (ppm)
Residual DNA content (qPCR)	≤1 ng/mg	n.a.	Informing analysis by Threshold immunoassay: ≤6.3 pg/mg	Informing analysis by Threshold immunoassay: ≤6.0 pg/mg	≤14.2 pg/mg
Protein purity (red. SDS-CE)	report result [%]	Main peak: 97.1% <Main peak: 1.8% >Main peak: 1.1%	Main peak: 98.2% <Main peak: 1.1% >Main peak: 0.7%	Main peak: 97.1% <Main peak: 2.0% >Main peak: 0.9%	Main peak: 98.1% <Main peak: 1.9
Contaminants					
Endotoxin (rFC assay)	≤30 EU/mg	n.a.	n.a.	n.a.	<0.16 EU/mg
Bioburden	≤10 CFU/10 mL	n.a.	n.a.	n.a.	0 CFU/10 mL
Test/analysis for characterization					
Test/analysis					
Nanoparticle size (Z_avg_)	report result	18.3 nm	18.5 nm	18.3 nm	18.8 nm
Nanoparticle size distribution (polydispersity index)	report result	0.04	0.03	0.04	0.03

**Table 6:** Logarithmic reduction factors (LRf) of individual removal/inactivation steps.

MODEL VIRUS.	X-MuLV	MMV
Detergent treatment	≥4.67	-
Anion exchange chromatography	≥3.59	2.25
Hydroxy apatite chromatography	0.75 ^(1)^	-
Nanofiltration	≥2.92 ^(2)^	≥5.40 ^(2)^
Total LRF	≥13.66 ^(2)^	≥7.65

^(1)^ LRFs not significant (<1) and therefore not included in calculation of total LRF. ^(2)^ Total LRF for X-MuLV was calculated using the logarithmic reduction factor of MMV for the nanofiltration step.

**Table 7:** Binding affinities (KD) of the top five clones and L20 BDS to mVRC01, P1B5, and 179NC75 mAbs measured by BLI.

Clone #	mVRC01 (KD)	P1B5 (KD)	179NC75 (KD)
Clone CL-5C9	4.37 × 10^−9^	3.75 × 10^−6^	4.53 × 10^−9^
Clone CL-3K21	1.74 × 10^−9^	3.98 × 10^−6^	3.16 × 10^−9^
Clone CL-9K21	3.86 × 10^−9^	4.56 × 10^−6^	3.46 × 10^−9^
Clone CL-3N15	3.87 × 10^−9^	1.37 × 10^−6^	4.22 × 10^−9^
Clone CL-4E22	3.06 × 10^−9^	2.28 × 10^−6^	2.54 × 10^−9^
L20 BDS	1.36 × 10^−11^	5.65 × 10^−8^	1.69 × 10^−11^

#: number

## Data Availability

The original contributions presented in this study are included in the article. Further inquiries can be directed to the corresponding authors.
